# Genome Comparisons of the Fission Yeasts Reveal Ancient Collinear Loci Maintained by Natural Selection

**DOI:** 10.3390/jof7100864

**Published:** 2021-10-14

**Authors:** Lajos Acs-Szabo, Laszlo Attila Papp, Matthias Sipiczki, Ida Miklos

**Affiliations:** Department of Genetics and Applied Microbiology, Faculty of Science and Technology, University of Debrecen, 4032 Debrecen, Hungary; papp.laszlo.attila@science.unideb.hu (L.A.P.); gecela@post.sk (M.S.)

**Keywords:** *Schizosaccharomyces*, gene order, synteny, genome evolution, rearrangement, natural selection

## Abstract

Fission yeasts have a unique life history and exhibit distinct evolutionary patterns from other yeasts. Besides, the species demonstrate stable genome structures despite the relatively fast evolution of their genomic sequences. To reveal what could be the reason for that, comparative genomic analyses were carried out. Our results provided evidence that the structural and sequence evolution of the fission yeasts were correlated. Moreover, we revealed ancestral locally collinear blocks (aLCBs), which could have been inherited from their last common ancestor. These aLCBs proved to be the most conserved regions of the genomes as the aLCBs contain almost eight genes/blocks on average in the same orientation and order across the species. Gene order of the aLCBs is mainly fission-yeast-specific but supports the idea of filamentous ancestors. Nevertheless, the sequences and gene structures within the aLCBs are as mutable as any sequences in other parts of the genomes. Although genes of certain Gene Ontology (GO) categories tend to cluster at the aLCBs, those GO enrichments are not related to biological functions or high co-expression rates, they are, rather, determined by the density of essential genes and Rec12 cleavage sites. These data and our simulations indicated that aLCBs might not only be remnants of ancestral gene order but are also maintained by natural selection.

## 1. Introduction

The genus *Schizosaccharomyces* consists of haplontic yeast species and belongs to the Taphrinomycotina subphylum of the Ascomycota phylum [1,2,3,4]. Their cells divide by medial fission and this is one of the most conspicuous features that set them apart from other yeasts. Besides, they show substantial similarity to metazoans in many important biological processes, even though their proteomes are more similar to higher eukaryotes in certain ways than to other fungal species [2,5,6,7]. These phenomena could be the consequences of their deep evolutionary origin [2,5]. The broadly known species of the genus is the *S. pombe*, which is a popular model organism of the cellular processes [8]. In the past few years this species has also become the subject of population genetic/genomic studies [9,10,11,12,13,14,15,16,17]. The genus comprises four additional species: *S. japonicus* [18,19,20,21,22,23,24,25], *S. octosporus* [26,27,28], *S. cryophilus* [29,30] and the recently described *S. osmophilus* [31]. The genus exhibits such evolutionary breadth that the idea of dividing the group into three genera has emerged several times in the past [32,33]. In spite of that, the gene content and structure are remarkably conserved among the fission yeasts species, higher than within the *Saccharomyces* or *Kluyveromyces* genera [2]. This was also supported by our previous work [30], where we assembled the large contigs of *S. cryophilus* to chromosome-like units using different in silico and molecular techniques. Comparison of the newly assembled genome to the genomic sequences of the related species revealed that numerous chromosomal rearrangements could have happened during the evolution of the species despite their conserved genomic structures [30]. Thus, the question has arisen: what could the reason for their unusually stable genome structures be?

Since high-quality genome sequences and annotations for almost every species in the genus are publicly available [2,34,35], we strongly believed that we might find the answers for the mentioned question through the comparison of their genome sequences and we hypothesized that the gene order is under the definite control of selection.

In our present study, we revealed the most conservative genomic regions of the species which might have been inherited from their last common ancestor. We investigated whether natural selection does favour certain gene orders or not and what the origin of gene orders in the fission yeasts could be. We compared the most conservative genomic segments to other regions by evolutionary rates of protein sequences and by intron gain and loss. We provide evidence that genes of certain GO categories tend to cluster to the most conservative regions of the genomes and examined some possible reasons for such clustering, too.

## 2. Materials and Methods

### 2.1. Species and Genomes Data

The genome sequences of the species used in this study are listed in Supplementary Table S1. Individual chromosome sequences with annotations were downloaded from NCBI with the following accession numbers: CU329670, CU329671 and CU329672 for *S. pombe*, KE503206, KE503207 and KE503208 for *S. octosporus*, KE546988, KE546989, KE546990, KE546991, KE546992, KE546993, KE546994, KE546995 and KE546996 for the contigs of *S. cryophilus* [2]. The annotated files were imported to the SnapGene Viewer software (version 5.3.2) (http://www.snapgene.com/products/snapgene_viewer/, accessed on 13 October 2021). In the case of the *S. cryophilus* and *S. octosporus* we used the improved chromosome structures and genome sequences provided by [30,35].

### 2.2. Whole Genome Alignments and Sequence Comparisons

Pairwise and multiple whole genome alignments were generated with the Mauve aligner (version 2015-02-26) using the progressiveMauve algorithm either with standard parameters or setting the option “use seed families” [36]. The minimum locally collinear block (LCB) weight was adjusted to 40 in all alignments initially after a few test runs. In the cases of finished pairwise alignments, the number of common LCBs were also estimated in Mauve by setting the desired length (LCB weight) in the software manually. Thus, the common number of LCBs at >1000 nt, >2000 nt, >3000 nt, >4000 nt, >5000 nt and >6000 nt were inferred in the case of distantly related Taphrinomycotina species pairs [37,38,39,40,41].

Whole genome dot plots were created with YASS (https://bioinfo.lifl.fr/yass, accessed on 13 October 2021) [42] using the whole genome sequences of the concerning species with the following parameters: *E* value: 1.0 × 10^−30^; X-drop: 50; window range: 100–200,000; window incr.: 2×; hit criterion: double and default parameters were used for the others. For the nucleotide comparisons we extracted the individual alignments in tabular form from the pairwise alignments in the cases of *S. japonicus*-*S. pombe*, *S. japonicus*-*S. octosporus* and *S. japonicus*-*S. cryophilus*. For the statistically most significant (*E* value: 0) alignments, the non-syntenic repetitive regions, such as 5S RNAs, tRNAs and high copy number genes were filtered out to avoid overestimation of genome conservation.

### 2.3. Genome Rearrangement Analyses

Rearrangement analyses were performed using either pairwise genomes or multiple genomes to estimate multi chromosomal distances (MCDs). Values of MCDs indicate an optimal number of rearrangement events possibly occurred in the genomes. In order to estimate that, the common collinear segments extracted from Mauve were submitted to GRIMM v2.01 as signed permutations (http://grimm.ucsd.edu/cgi-bin/grimm.cgi, accessed on 13 October 2021) [43].

### 2.4. Orthology Inference

Protein sequences of *S. pombe* were used (as its genome is the most refined and well-studied) to identify the putative orthologues of *S. japonicus*. BLASTp [44] search was performed in the website of EnsembleFungi (https://fungi.ensembl.org/index.html, accessed on 13 October 2021) with the following parameters: *E* value: 1.0 × 10^−3^; matrix: BLOSUM62 or BLOSUM45 and default parameters were used for the others. In order to find the best hit and to avoid missing any possible orthologues, gene neighbourhoods were also considered in the orthology inference. That is, when the BLASTp search identified two (or more) possibilities as proper hits, that gene was accepted as an appropriate one which had orthologous adjacent genes (syntenic genes). Besides, gene adjacency also contributes to the identification of genes which exhibit low sequence similarity (for example hypothetical genes). Thereafter the analyses were extended to the other two fission yeast species using our previously created dataset [30].

For the identification of the putative orthologues of *S. pombe* and *S. japonicus* protein sequences in other fungal species (Supplementary Table S1), BLASTp searches were performed in the database of the Broad Institute in the cases of *Cryptococcus gattii* [45,46], *Meyerozyma guilliermondii* [47], *Pneumocystis murina* [39], *Aspergillus nidulans* [48], *Neurospora crassa* [49] and in the database of the DOE Joint Genome Institute in the cases of *Rhizopus oryzea* [50], *Ustilago maydis* [51], *Yarrowia lipolytica* [52], *Debaroymyces hansenii* [53], *Botrytis cinerea* [54], *Taphrina deformans* [37], *Saitoella complicate* [38] with the following parameters: *E* value: 1.0 × 10^−3^–1.0 × 10^−5^; matrix: BLOSUM62 or BLOSUM45 and default parameters were used for the others. Reciprocal BLASTp analyses were also carried out for the most reliable results.

### 2.5. Visualization of Collinear Blocks

Small-scale collinear blocks were depicted with the online tool Simple Synteny (https://www.dveltri.com/simplesynteny/, accessed on 13 October 2021) [55]. Genome-scale collinearity were displayed using the OrthoClusterDB online platform with the following parameters: order and strandedness: -r -s, synteny block size lower bound: 2, upper bound: 2000 and default parameters were used for the others (http://genome.sfu.ca/cgi-bin/orthoclusterdb/runortho.cgi, accessed on 13 October 2021) [56].

### 2.6. Phylogenetic Tree Constructions

Certain protein sequences were concatenated and aligned either with MAFFT v. 7.221 (http://mafft.cbrc.jp/alignment/server/, accessed on 13 October 2021) [57] or MUSCLE (http://www.ebi.ac.uk/Tools/msa/muscle/, accessed on 13 October 2021) [58,59]. In the case of MAFFT, E-INS-i strategy was used. The curation of the MUSCLE alignments was done with Gblocks (http://molevol.cmima.csic.es/castresana/Gblocks_server.html, accessed on 13 October 2021) [60]. The multiple alignments were used for phylogenetic tree constructions either with the Neighbour-Joining (NJ) algorithm available at: (http://mafft.cbrc.jp/alignment/server/phylogeny.html, accessed on 13 October 2021) using the JTT substitution model or the Maximum Likelihood (ML) algorithm PhyML 3.0 available at: (http://www.atgc-montpellier.fr/phyml/, accessed on 13 October 2021) [61]. In the case of NJ, heterogeneity among sites were estimated. Branch supports were estimated from bootstrap analyses (100 replications). For the PhyML analysis, the LG substitution model was chosen. Model selection for the analysis was conducted by SMS (http://www.atgc-montpellier.fr/phyml/, accessed on 13 October 2021) [62]. The number of substitution rate category was adjusted to 4, gamma distribution parameter was estimated and the proportion of invariable sites was fixed to 0. Branch support was estimated with approximate likelihood ratio test (aLRT SH-like) [63].

The created trees were displayed with FigTree v1.4.2 (http://tree.bio.ed.ac.uk/software/figtree/, accessed on 13 October 2021) or with Archaeopteryx (https://sites.google.com/site/cmzmasek/christian-zmasek/software/archaeopteryx, accessed on 13 October 2021) [64].

### 2.7. Modelling Genome Evolution

To ascertain that the collinear blocks are consequences of natural selection rather than just remnants of ancestral gene order due to incomplete genome reshuffling, two different analyses were performed. First, we tested the effects of neutral evolution modelled by simple chromosomal changes with a custom Python script. We created a root genome with 5000 genes represented as unsigned permutations. Then, we rearranged that root genome for certain times at random sites using the estimated data of MCDs (590 for *S. pombe*; 592 for *S. cryophilus* and 598 for *S. octosporus*). In simple words, we transformed the fictive *S. japonicus* genome to *S. pombe*, to *S. octosporus* and to *S. cryophilus.* Further description of the modelling parameters is available in the Supplementary Information. Python scripts developed for this study are available at Github: https://github.com/Laci01/Laci01/tree/Schizosaccharomyces_synthetic, accessed on 13 October 2021.

We also utilized the Artificial Life Framework (ALF) [65]. We used the standalone version of the ALF, only the parameter file was generated at the website (http://alfsim.org/#index, accessed on 20 February 2021). Evolutionary reference unit was adjusted to substitutions per site. Root genome was randomly generated with 5000 proteins, minimum protein length was 25 amino acids with a gamma length distribution of (k, Ɵ) (2.4019, 133.8063). Block size was 1 aa. We used the following tree as a custom species tree in Newick format: ((Sp:0.12306018000000002,(Sc:0.03208717999999999,So:0.04437972000000001):0.12928902999999997):0.232529725,Sj:0.232529725). For the sequence types we used the preset “WAG, Zipfian gaps”, only the substitution model was adjusted to LG. “Inversion and translocation only” option was chosen from the genome rearrangement (genome level events) setup with the following parameters: rate of inversion: 0.13; maximum inversion length: 300; rate of translocation: 0.13; maximum translocation length: 300; rate of inverted translocation: 0.5. A detailed description of the modelling parameters is available in the Supplementary File S1.

### 2.8. Study of Evolutionary Rates and Intron Loss/Gain

The dataset of evolutionary rates of the fission yeasts protein sequences was obtained from [2]. They established the evolutionary rates of 4220 1:1:1:1 putative orthologous proteins. For the investigation of intron loss and gain, sources of [66] were used. They investigated 2963 1:1:1:1 orthologous genes, among which 2108 intron containing genes were found. They observed 1775 conserved intron positions and 808 unique intron positions. Evolutionary rates and intron loss/gain of the genes in the ancestral locally collinear blocks (aLCBs) were established and compared to the relevant values of other genes outside of the aLCBs.

### 2.9. GO Enrichment Analyses

Gene ontology categories and the corresponding gene sets of the fission yeasts biological processes were downloaded from the database of Pombase (https://www.pombase.org/browse-curation/fission-yeast-go-slim-terms, accessed on 12 February 2020) [67]. At that time there were 728 genes from the 5141 *S. pombe* genes which had no GO annotations. Only the 53 main GO categories were considered for the analysis. If a gene was represented in multiple categories, it was assigned to each. Mitochondrial genes, rRNAs and tRNAs were not included. Genomic localisations of the genes from the different GO categories were established and associations with the aLCBs were counted.

### 2.10. Density of Essential Genes and Rec12 Cleavage Sites

Essential genes are defined as genes that cause cell death when they are knocked-out. Quantifications of density of essential genes were performed by examining the gene deletion viability of *S. pombe* genes that were available in the Pombase database (https://www.pombase.org/downloads/phenotype-annotations, accessed on 12 February 2020). At that time, there were 4899 genes from the 5141 *S. pombe* genes which had information about their deletion viability. Genes whose deletion viability is condition dependent also counted as essential genes.

Rec12 (Spo11 in *S. cerevisiae*) is a topoisomerase-related protein which initiates recombination by forming developmentally programmed DNA double-strand breaks. To establish the localizations of experimentally verified Rec12 cleavage sites of *S. pombe,* datasets of [68] were used (603 sites).

### 2.11. Analyses of Co-Expression Rates

Values of co-expression rates are originated from [69], where the authors assembled the *S. pombe* co-expression network which is based on 9 independent expression datasets. The co-expression network contains information on 5063 *S. pombe* genes.

### 2.12. Normalisation and Randomisation

Normalisation of the data were performed by dividing the concerning values by their mean values. Randomisation and its statistical evaluation was performed as described in [70]. Random numbers were generated at the website of Random.org (https://www.random.org/integers/, accessed on 13 October 2021), as it offers true random numbers which come from atmospheric noise.

### 2.13. Statistical Analyses

Normal distributions of the data were tested by Shapiro-Wilk and Anderson-Darling tests. Single-case t-probe was used in the case of “one to many” comparisons. Related pairwise data was tested using Mann-Whitney U test. Multiple normally distributed data was tested by one-way ANOVA test or repeated-measures ANOVA (RM-ANOVA) test. For datasets that proved not to be normally distributed, Kruskal-Wallis test were used for multiple comparisons followed by pairwise Dunn test as post hoc tests. Equal distributions were tested by Kolmogorov-Smirnov test. Correlation of the data was tested by linear Pearson correlation test or Spearman correlation test. *p* values were considered significant below the alpha level 0.05. Bonferroni corrections were used to minimalize the effect of Type I error. All statistical analyses were performed using the Past3 program (http://folk.uio.no/ohammer/past/, accessed on 7 January 2021) [71] and Microsoft Office Excel 2016.

### 2.14. Image Creation

All images used in this study were created with their corresponding software: Mauve [36], YASS [42], FigTree, SimpleSynteny [55], OrthoclusterDB [56], Past3 [71], Microsoft Office Excel 2016 and PowerPoint 2016. Modification of the images such as labelling, positioning or highlighting was undertaken in Microsoft Office PowerPoint 2016, Paint.net v4.2.6 and InkScape v1.0.2. All images created in this study represent the original data; modifications which alter the real values were not made.

## 3. Results

### 3.1. Structural- and Sequence Evolution of the Fission Yeasts Show Unequivocal Correlations despite Their Evolutionary Breadth

**Figure 1:**
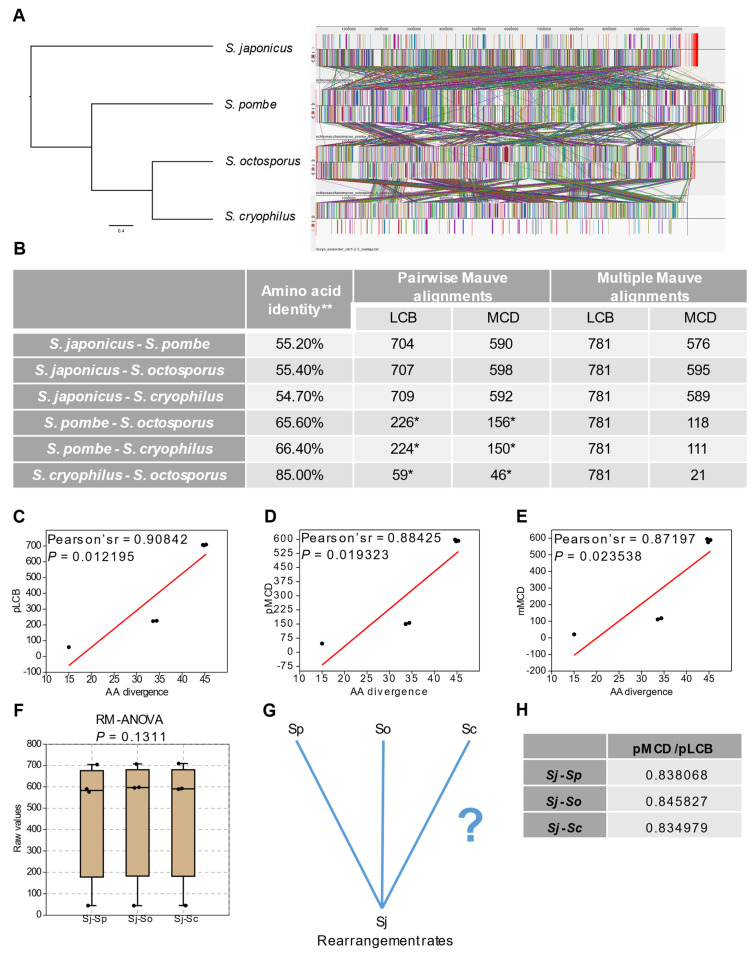
Sequence and structural evolution of the fission yeasts are correlated. (**A**) Whole genome alignments of the fission yeasts using *S. japonicus* as reference genome. Colourful rectangles and lines represent locally collinear blocks (LCBs) which are the most conserved collinear regions of the genomes. (**B**) Overall amino acid identity and the number of LCBs and corresponding multi-chromosomal distances (MCDs) in pairwise and multiple scenarios. * data are originated from [30]. ** data are established by [2]. (**C**–**E**) Correlations between amino acid differences (aa diff) and pairwise LCBs (pLCB), pairwise MCDs (pMCD), multiple MCDs (mMCD). Black dots represent the fission yeast species pairs, red lines are regression lines. The concerning values are correlated significantly in all pairwise comparisons. (**F**) Statistical evaluation of the aforementioned data in respect of the *S. japonicus* lineage. The data indicated that there were no significant discrepancies among the species pairs. (**G**,**H**) Rearrangement rates (pMCD/pLCB) suggested that the other fission yeasts species are almost equally distant from *S. japonicus*.

Our previous results suggested that structural- and sequence evolution of *S. pombe*, *S. octosporus* and *S. cryophilus* are correlated [30]. Here, we wanted to learn whether this phenomenon is true for the *S. japonicus* lineage, too. Pairwise and multiple whole genome alignments created with the Mauve aligner [36] indicated conserved, albeit highly rearranged genome structures (Figure 1A and Supplementary Figure S1). These findings were also supported by GRIMM rearrangement analyses [43] performed on the extracted locally collinear blocks (LCBs) (Figure 1B). LCBs are conserved collinear regions of the genomes identified by sequence similarity. We compared the values of LCBs and multi chromosomal distances (MCDs) to the data of amino acid differences (aadiff) [2] which were based on more than 2000 single-copy orthologous protein sequences (Figure 1B). We performed correlation analyses on the data concerning every species pairs (Figure 1C–E) and all the three comparisons (aadiff-pairwise LCBs, aadiff-pairwise MCDs and aadiff-multiple MCDs) seemed to correlate significantly (Pearson’s *r* = 0.90807, 0.88474, 0.87197, *p* = 0.012287, 0.019163, 0.023538, respectively) which may indicate that sequence- and structural evolution of the fission yeasts are correlated. For further statistical evaluation in the case of the *S. japonicus* lineage, we performed analysis of variance tests on the pairwise data and on the normalised data, too (Figure 1F and Supplementary Figure S2). The tests showed no significant differences among the values (RM-ANOVA, *p* = 0.1311 and *p* = 0.1988, respectively), which suggest that the other three fission yeast species are almost equally distant from *S. japonicus* regarding chromosome rearrangements (Figure 1F,G and Supplementary Figure S2). This idea was also confirmed by the estimations of structural change rates (MCDs/LCBs): *S. japonicu*-*S. pombe*: 0.838; *S. japonicu*-*S. octosporus*: 0.846; *S. japonicus*-*S. cryophilus*: 0.835 (Figure 1G,H).

### 3.2. Extent of Whole Genome Conservation of the Fission Yeasts Is Almost Equal

**Figure 2:**
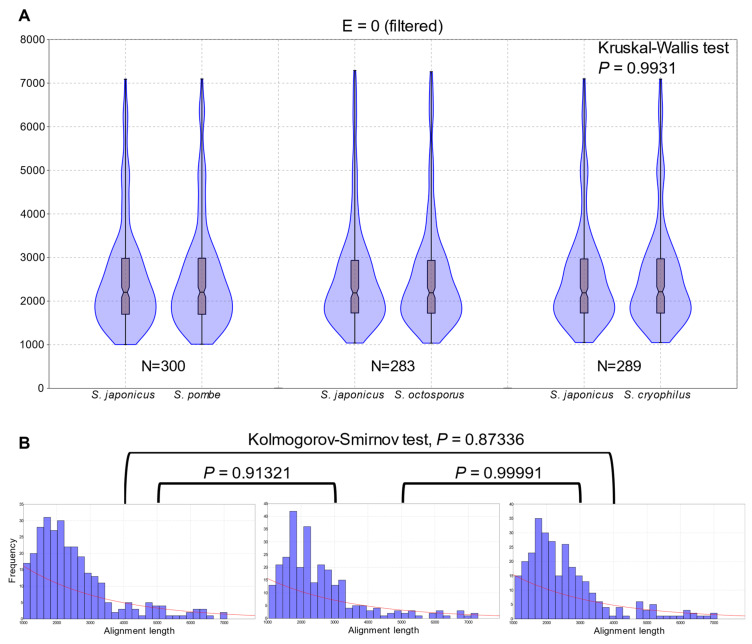
Conserved regions of the genomes show equal distributions. (**A**) Length distribution of the pairwise whole genome alignments created with YASS (*E* = 0). Violin plots show kernel density for the samples. Box plots indicate the 25–75 percent quartiles. Horizontal lines within the boxes show the medians of the samples, notches indicate the 95 percent confidence intervals for the medians. Minimal and maximal values are depicted by the whiskers. N: sample size. *Y*-axis shows the lengths (in nucleotides) of the individual alignments. There was no significant difference in the variance of the data. (**B**) Histograms depicting the most conserved genomic regions of *S. japonicus* in different pairwise scenarios and their divergences from the concerning exponential distributions. Bin = 30. The distributions of the pairwise cases were not significantly different.

Since the aadiff and the structural changes of the fission yeast species indicated a nearly uniform divergence from the *S. japonicus* lineage (Figure 1H), we wanted to examine their genome conservation at the nucleotide level, too. To establish the extent of genome conservations relative to *S. japonicus*, we created pairwise whole genome dot-plots with YASS [42] (Supplementary Figure S3). Thereafter, we extracted the list of alignments from all the three pairwise alignments and we examined the statistically most significant ones (*E* value = 0) and less stringent alignments (*E* value ≤ 1 × 10^−30^) (Supplementary Tables S2–S4), too. We filtered out the non-syntenic repetitive regions (for example 5S rDNAs) and other multi occurring sites from the most stringent alignments (*E* value = 0); moreover, we considered only those alignments that exceeded 1000 nucleotides in size to avoid overestimation of genome conservations (Figure 2A) (Supplementary Tables S5–S7). In that particular way, we found 283–300 individual genome segments which comprised the 6.4–6.8% of the whole genomes (Supplementary Table S8). These data may indicate a uniform conservation of the genomes from the *S. japonicus* perspective. Although the overall sizes of the most conserved regions might seem implausible, we should bear in mind that the diversity at the nucleotide level always exceeds the diversity of the protein sequence level. We compared the number and the extent of the pairwise alignments to each other and we observed only slight but not significant differences among the concerning values (Kruskal-Wallis test, *p* = 0.9931) (Figure 2A). For an alternative approach, we analysed the distributions of the pairwise alignments and none of the comparisons proved to be significantly different (Kolmogorov-Smirnov test, *p* = 0.99991–0.87336) (Figure 2B–D). Evaluation of the less stringent alignments (*E* value ≤ 1 × 10^−30^) showed similar tendencies to the stricter alignments, but their extent comprised more than 30% of the genomes (Supplementary Figure S4 and Supplementary Table S8).

Thus it seems that the extents of genome conservation are approximately the same among the different comparisons when we use *S. japonicus* as reference. In addition to that, thorough visual inspections of the Mauve alignments, dot plots and examinations of the localisations of alignments indicated that the same genomic segments of the concerning species formed collinear blocks in numerous cases.

### 3.3. Analyses of Gene Level Genome Conservation Reveals Ancient Collinear Loci Inherited from the Last Common Ancestor of the Fission Yeasts

Although the whole genome alignments previously created with YASS and Mauve suggested that numerous common LCBs shared by the four species may exist, further analyses were required for a gene level resolution. As a first step we inferred all of the orthologues between *S. pombe* and *S. japonicus*. After that we extended our analysis to *S. octosporus* and to *S. cryophilus* based on our previous results [30] and we created a database that contains most of the putative orthologues of the species (Supplementary Table S9).

**Figure 3:**
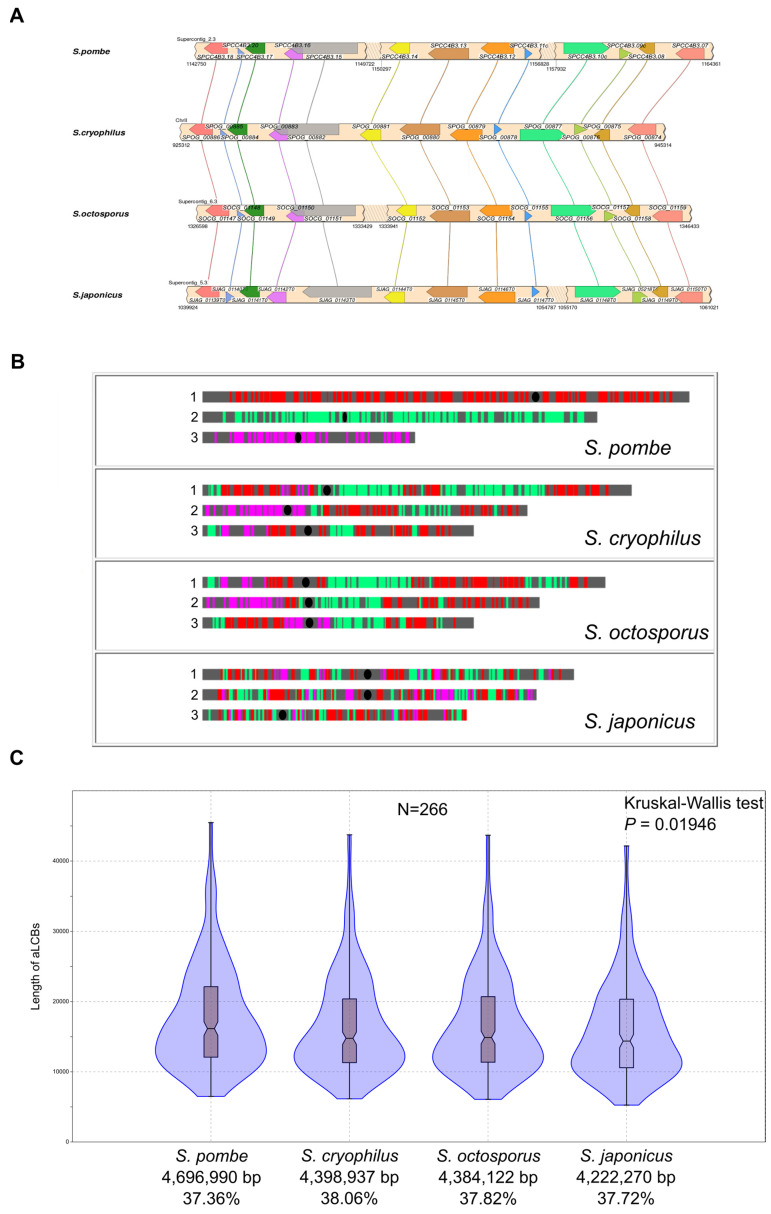
Ancestral locally collinear blocks (aLCBs) of the fission yeasts inherited from their last common ancestor. (**A**) Depiction of a common aLCB from the genomes of the four species. All the genes in the concerning regions were in the same order and orientation. (**B**) Chromosomal localisations of the aLCBs in the species using *S. pombe* as reference. As was expected, *S. japonicus* exhibited the most disperse localisation of aLCBs along its chromosomes. Black ellipses indicate centromere positions. (**C**) Length distributions of the inferred aLCBs within the species. Violin plots show kernel density for the samples. Box plots indicate the 25–75 percent quartiles. Horizontal lines within the boxes show the medians of the samples, notches indicate the 95 percent confidence intervals for the medians. Minimal and maximal values are depicted by the whiskers. *n*: sample size. Values under the species names are the overall lengths of aLCBs and the percentages comparing to the sizes of the whole genomes. As the sizes of the aLCBs included the intergenic regions between coding regions, too, values of *S. pombe* and *S. japonicus* were proved to be significantly different. Pairwise statistics (Kolmogorov-Smirnov tests) are presented in Supplementary Figure S5.

Thereafter, we selected LCBs that consist of at least five orthologous genes [72] in the same orientation and order relative to each other in all of the species (Figure 3A). If there were any changes, such as gene insertion or gene deletion, in those blocks in even one species, we did not consider it further as a LCB (Supplementary Table S9). These segments are ancient loci, as the species likely inherited them from their last common ancestor, so hereafter we refer to them as ancestral-LCBs (aLCBs).

Study of localisation of the aLCBs showed that aLCBs could not be found or were not common in the subtelomeric regions (Figure 3B and Supplementary Table S9). In other parts of the chromosomes we found 266 aLCBs with a remarkable mean value of 7.73 genes/blocks (Figure 3B and Supplementary Table S10). Those aLCBs included 2055 genes which are ~40–42 % of the whole gene contents and their overall lengths (including the intergenic regions between the coding sequences) comprise 37–38% of the genomes in each *Schizosaccharomyces* (Figure 3C). Comparison of the lengths of aLCBs showed significant differences in the variance (Kruskal-Wallis test, *p* = 0.01946 followed by Dunn’s post hoc test, *p* = 0.01178) and in the distribution (Kolmogorov-Smirnov test, *p* = 0.023497) in the case of *S. pombe*-*S. japonicus* (Figure 3C and Supplementary Figure S5). The reason for these phenomena is a substantial difference in the intergenic sequence lengths among the species [73].

### 3.4. The aLCBs Are Not Only Remnants of Ancestral Gene Order but Are Also Maintained by Natural Selection

We wanted to find out whether the existence of aLCBs is a consequence of selection or of chance. As 40–42% of the genes of all fission yeasts are located at those aLCBs, we could easily minimize the possibility of chance. Instead, we asked whether these aLCBs are just remnants of ancestral gene order due to incomplete reshuffling or whether they are maintained by natural selection.

In order to address these questions, we performed a series of synthetic genome evolution with two different approaches. First, we tested the effects of a neutral evolution modelled by simple chromosomal changes and second, we used the Artificial Life Framework (ALF) pipeline [65] for a more sophisticated approach. In the first model, changes occur without any restriction; in contrast, ALF evolves the synthetic genomes along a specific phylogenetic tree. If we make the assumption that genomes evolve in a neutral way and are not under the control of selection, then the simulated data should be quite similar to that observed in the real genomes.

We performed 100 independent simulations with a custom Python script and we also created 100 simulations with ALF (see Section 2 and Supplementary File 1 for detailed descriptions of the simulations). We searched for aLCBs in the synthetic genomes that we found in the real genomes (Table 1A). The results of the random simulations were significantly different compared to the data of the real genomes (Table 1B). They differed in the number of aLCBs, in the sum of genes located to those aLCBs and even in the mean number of genes/blocks (Single-case *t*-probes, *p* = 1.53 × 10^−83^, 7.36 × 10^−95^, 1.24 × 10^−5^, respectively) (Table 1A). Slightly different results came from the analyses performed with the ALF (Table 1A). The number of found aLCBs showed no significant discrepancy compared to the real value (Single-case *t*-probes, *p* = 0.44274) which also indicated that the used rearrangement rates were well estimated (Table 1B). However, the values of the sum of genes and of the mean number of genes/blocks were significantly different (single-case *t*-probes, *p* = 0.00016125, 3.33 × 10^−14^, respectively) (Table 1B). These results support the idea that the aLCBs observed in the genomes of fission yeast are not only remnants of ancestral gene order, but might also be under the control of selection.

### 3.5. Gene Order of the aLCBs Is Mainly Fission-Yeast-Specific but Further Supports the Idea of Filamentous Ancestors

If the aLCBs in the genomes of the fission yeasts are not only remnants of ancestral gene order but are also maintained by natural selection, there may be a slight chance that these certain gene orders can be observed in other species, too. Since whole genome sequences of other Taphrinomycotina species (*Taphrina deformans, Saitoella complicata, Pneumocystis murina, Protomyces lactucae-debilis, Neolecta irregularis*) have become available [37,38,39,40,41] (Supplementary Table S1), comparative analyses can be performed with phylogenetically less distant species.

**Figure 4:**
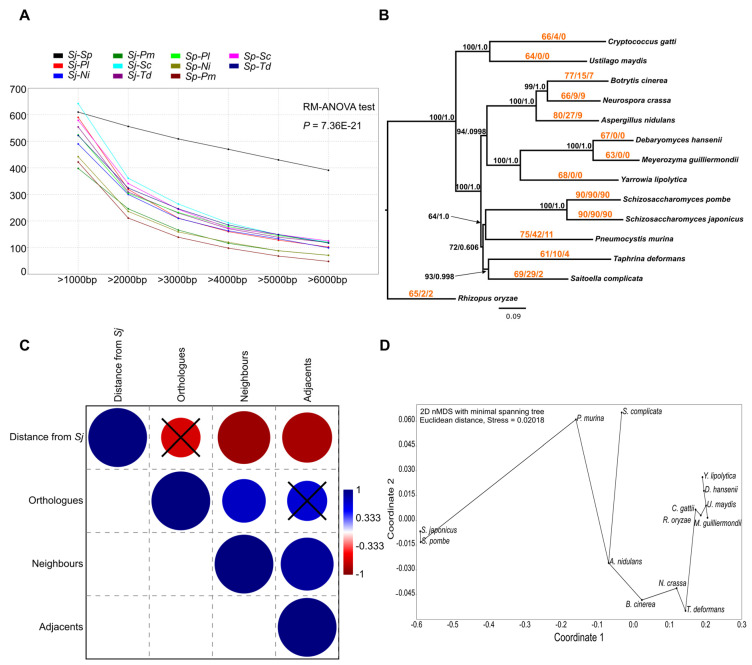
Gene order of the aLCBs is mainly fission-yeast-specific but further supports the idea of filamentous ancestors. (**A**) Frequency graph of the common LCBs among the species pairs in different lengths. The figure shows that none of the Taphrinomycotina species exhibit such high number of common LCBs with the fission yeasts than the *S. japonicus*-*S. pombe* species pairs. Abbreviations: Sj-*S. japonicus*; Sp-*S. pombe*; Pl-*P. lactucae-debilis*; Pm-*P. murina*; Td-*T. deformans*; Sc-*S. complicata*; Ni: *N. irregularis*. (**B**) Phylogenetic tree inferred from 26 common putative orthologous protein sequences from 11 randomly selected aLCBs of the fission yeasts. Colourful numbers on the branches indicate the following data: (number of common orthologues with the *Schizosaccharomyces*/number of neighbouring genes among the orthologues/number of adjacent genes among the orthologues). Statistical support of the nodes came from NJ bootstrap analyses (100 replicates) and ML aLRT. (**C**) Pairwise Spearman correlations of the concerning data. Blue circles represent positive correlations, reds indicate negative correlations. Phylogenetic distance from *S. japonicus* and the number of common orthologues, moreover the number of common orthologues and the number of adjacent orthologues were not correlated significantly (black crossed circles). Phylogenetic distance and the found neighbouring genes and adjacent genes were correlated significantly. Bonferroni corrected *p* values are presented in Supplementary Table S13. (**D**) 2D nMDS with minimal spanning tree computed from the previous data shows that gene order of the fission yeast exhibits more similarity to the filamentous fungi than to the other yeast species.

Thus, we carried out pairwise whole genome alignments using both *S. japonicus* and *S. pombe* as reference (Supplementary Figures S6 and S7). Our findings coincided with others [37,39] as no long range collinear regions can be found among the species. Then, we compared the number of common LCBs (inferred by Mauve) at different lengths (see methods) among the species pairs. As it was expected, none of the non-*Schizosaccharomyces* species exhibited relevant numbers of common LCBs compared to the fission yeasts regardless of the sizes of the LCBs (RM-ANOVA, *p* = 7.36 × 10^−21^) (Figure 4A).

Since we are aware of the fact that substantial phylogenetic distance can negatively affect the efficiency of DNA-based alignments, we implemented a thorough gene level analysis (using their protein sequences) with 11 randomly chosen aLCBs, which contained 90 genes overall. We also extended the list of species with nine additional fungi from other subphyla [45,46,47,48,49,50,51,52,53,54] (Supplementary Table S1). The numbers of the found putative orthologues ranged between 61 and 80 across the species (Supplementary Table S11). We used the concatenated protein sequences of 26 common orthologues with 6210 well-aligned sites to construct a phylogenetic tree as we wanted to find out whether the number of observed orthologues and the phylogenetic positions of the species are related or not (Figure 4B) (Supplementary Table S12). Although it was broadly true that the phylogenetically distant species shared a smaller number of putative orthologues with the fission yeasts, the correlation was not significant (Spearman’s *rs* = −0.66079, *p* = 0.060534) (Figure 4C) (Supplementary Table S13).

**Figure 5:**
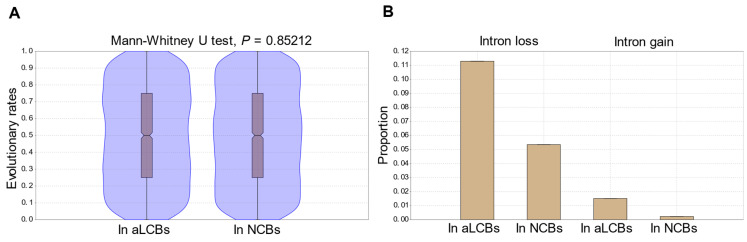
Sequence- and gene-structural changes are not different between the aLCBs and the other parts of the genomes. (**A**) Distribution of evolutionary rates of proteins whose genes were located at the aLCBs and outside of aLCBs (NCBs). Violin plots show kernel density for the samples. Box plots indicate the 25–75 percent quartiles. Horizontal lines within the boxes show the medians of the samples, notches indicate the 95 percent confidence intervals for the medians. Minimal and maximal values are depicted by the whiskers. The values are not significantly different. (**B**) Bar charts depict the proportion of genes in the aLCBs and outside of aLCBs (NCBs) which showed intron loss and gain in their sequences.

For the examination of the localization of orthologous genes we considered two scenarios as we counted the number of genes which were situated in each other’s neighbourhood (maximum five intersecting genes) and the number of genes which were adjacent (Figure 5B). We noticed that the numbers of neighbouring and adjacent genes were consistent with the phylogenetic positions of the species (Spearman’s *rs* = −0.89217 and −0.84583, *p* = 0.000108 and 0.000827, respectively) (Figure 4C) (Supplementary Table S13).

These results coincided with the expectation that the observed gene order of aLCBs was mainly fission-yeast-specific. Nevertheless, we have to consider that the Pezizomycotina species shared more common adjacent orthologous genes with the fission yeasts than others did (except *P. murina*). Thus, there is a slight chance that the observed adjacency of genes might be remnants of an ancient filamentous gene order (Figure 4D).

### 3.6. Comparisons of Sequence- and Gene Structural Changes Indicate That Gene Sequences in the aLCBs Are as Mutable as Any Sequences in Other Parts of the Genomes

Since the aLCBs in question are ancient loci maintained by natural selection in terms of gene order, we wanted to find out whether the aLCBs exhibit higher conservation at their sequence level, too. There is great deal of evidence confirming the assumption that structural (gene order) and nucleotide evolution depend on two different molecular clocks [74]. Thus, it is unreasonable for a chromosomal segment which remains unchanged in gene order to also remain unchanged in nucleotide sequences. However, rearrangements or any kind of changes that alter gene order could be mutagenic, thus, sequences which did not have to undergo events of this kind might be more conservative than other parts of the genome sequences.

In order to examine this, we performed two different analyses. First, we compared the evolutionary rates of protein sequences whose genes are located at the aLCBs and outside of the aLCBs. Second, we compared the intron structural changes of the genes, to obtain information about the amount of intron gain and loss in and outside the aLCBs.

Although the analyses of protein sequences do not necessarily reflect all the changes that possibly occur in the concerning DNA sequences (synonymous mutations, homoplasy), we were still able to obtain an idea of their evolution, especially of the speed of their evolution. We used the data of [2] and we found a slight but not significant difference (Mann-Whitney U test, *p* = 0.85212) between the evolutionary rates of proteins localised to the aLCBs and to other parts of the genomes (Figure 5A, Supplementary Figure S8).

Next, we examined the gene structures in terms of intron loss and gain and their distribution along the genomes. We used the dataset provided by [66]. Examination of the unique intron positions revealed 232 and 31 genes within aLCBs and 165 and 7 genes outside of aLCBs which showed intron loss and intron gain, respectively (Figure 5B). Although the data above seem to be quite surprising, we should bear in mind that a precise identification of intron loss/gain event depends on strict orthology inference and synteny to exclude false results. Therefore, the sampled gene sets from the study of Zhu and Niu [66] largely overlapped with the genes from the aLCBs. However, if we consider that *S. pombe* has 2512 intron containing genes according to Pombase and 2108 genes were sampled from that pool by Zhu and Niu [66] then the result could be quite representative.

These results indicate that the gene sequences situated in the aLCBs are not more conserved than the genes situated in other part of the genomes.

### 3.7. Genes of Certain GO Categories Tend to Cluster to the aLCBs

**Figure 6:**
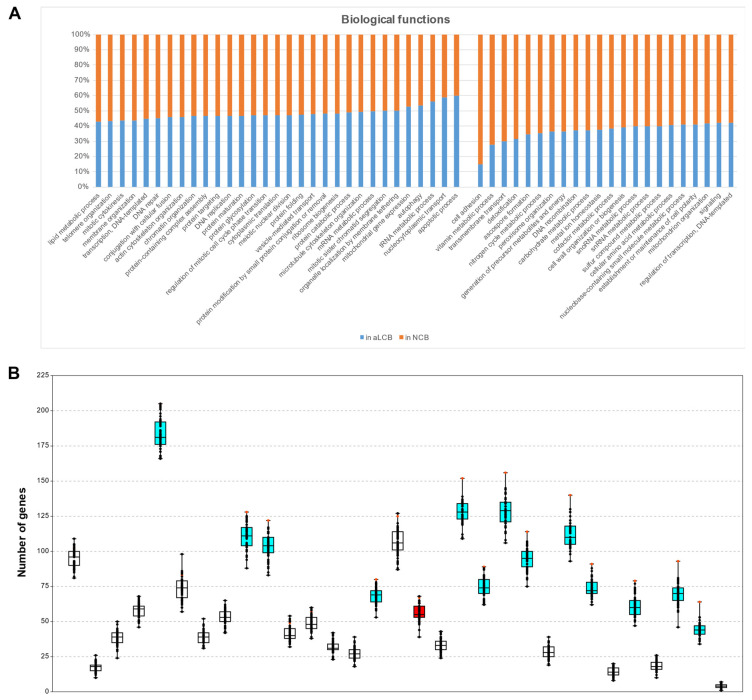
Genes of certain GO categories tend to cluster to the aLCBs. (**A**) Proportion of genes from different GO categories situated in the aLCBs and outside of aLCBs (NCBs). The first 31 categories (from left to right) are overrepresented in the aLCBs. (**B**) Data of 50 random sets of the 31 GO categories whose genes tend to cluster to the aLCBs (order of the GO categories is the same as above). Red dots on the whiskers show the real values observed in the genome of *S. pombe*, black dots indicate the values of the random sets. Real values are significantly different from the random sets in all the cyan and red coloured boxes according to the randomisation *p* values. Cyan colours indicate those categories which remained significant after the single-case *t*-probes, too. Red colour shows the only GO category (meiotic nuclear division) which showed significant clustering according to the randomisation *p*, but not after the single-case *t*-probe. There were 13 GO categories whose genes showed significant enrichment in the aLCBs.

We examined what kinds of genes are located at the aLCBs in terms of biological functions. We downloaded the gene lists of the 53 GO slim terms of biological processes from Pombase and established their distributions along the chromosomes of *S. pombe*. Since there is no specific information on the GO categorisation of genes for the other fission yeast species, we examined only the *S. pombe* data in the following. According to our data, 2055 genes from the 5141 (39.97%) of the whole gene content of *S. pombe* are situated in the aLCBs. However, if we did not consider those genes that had no information about their annotations (728 genes), then the percentage became slightly different: it was elevated from 39.97% to 42.07%. If we suppose that the genes in the genomes are randomly distributed, the members of the GO categories should not exceed that 42.07% in the aLCBs. If they do, it could either result from chance or could be an overrepresentation for some reasons. We found that 31 groups were overrepresented in the aLCBs (Figure 6A and Supplementary Table S14). To ascertain whether the enrichment of these GO terms were statistically significant or not, we produced 50 random sets and examined the localization of the genes belonging to the different GO terms (Figure 6B and Supplementary Table S15). After that, 14 out of the 31 categories proved to be significant (Table 2 and Supplementary Table S15). For certainty, we compared the real data to the values of the random sets with single-case *t*-probes and 13 out of the 14 remained significant, such as chromatin organisation, nucleocytoplasmic transport and ribosome biogenesis for instance (Figure 6B, Table 2 and Supplementary Table S15).

Although genes from certain GO terms are significantly enriched in the aLCBs, those genes still show disperse localisations on the chromosomes. For example, the genes of the GO term apoptotic processes localised in all the three chromosomes of *S. pombe*: four genes in ChrI, four genes in ChrII and two genes in ChrIII, but all the genes are a substantial distance from each other. Thus, the observed overrepresentation of certain GO groups is not a consequence of physical proximity. Maybe the precise coregulation of the concerning genes could be the reason for such clustering.

### 3.8. Co-Expression Rates of the Genes in the aLCBs Are Not Higher Than the Co-Expression Rates of Other Genes Outside the aLCBs

Since we indicated that the genes from the same GO categories are not colocalised within the aLCBs, we wanted to examine the co-expression patterns of the neighbouring genes. We used the data of Koch et al. for the analyses [69]. First, we measured the mean value of co-expression rates among genes in the aLCBs, which turned out to be 0.0408 (Table 3). Then, we randomly selected blocks of adjacent genes from outside of the aLCBs (mean co-expression: 0.0394) and compared the data to the values originated from the aLCBs (Table 3). We observed just a slight but not significant difference between the values (Mann-Whitney U test, *p* = 0.86051) (Table 3.) These data indicate that genes within the aLCBs do not tend to be co-expressed in higher rates than the genes localised outside of the aLCBs. However, if we compared the above values to the mean co-expression value of the whole genome (0.0287) then the latter value shows a significantly lower rate (Kruskal-Wallis test, *p* = 3.787 × 10^−33^).

### 3.9. GO Enrichment in the aLCBs Is Not Related to the Biological Functions, It Is Rather Determined by the Density of Essential Genes and Rec12 Cleavage Sites

For the next step, we wanted to determine some possible reasons for such positioning of the genes from certain categories. Pál and Hurst provided evidence for the coevolution of gene order and recombination rates, in context of which they also reported that essential genes cluster into regions of low-recombination in the genome of *S. cerevisiae* [75]. Thus, we examined the positions of experimentally verified Rec12 cleavage sites [68] and the distribution of essential genes in the genome of *S. pombe* in context with the GO categories.

**Figure 7:**
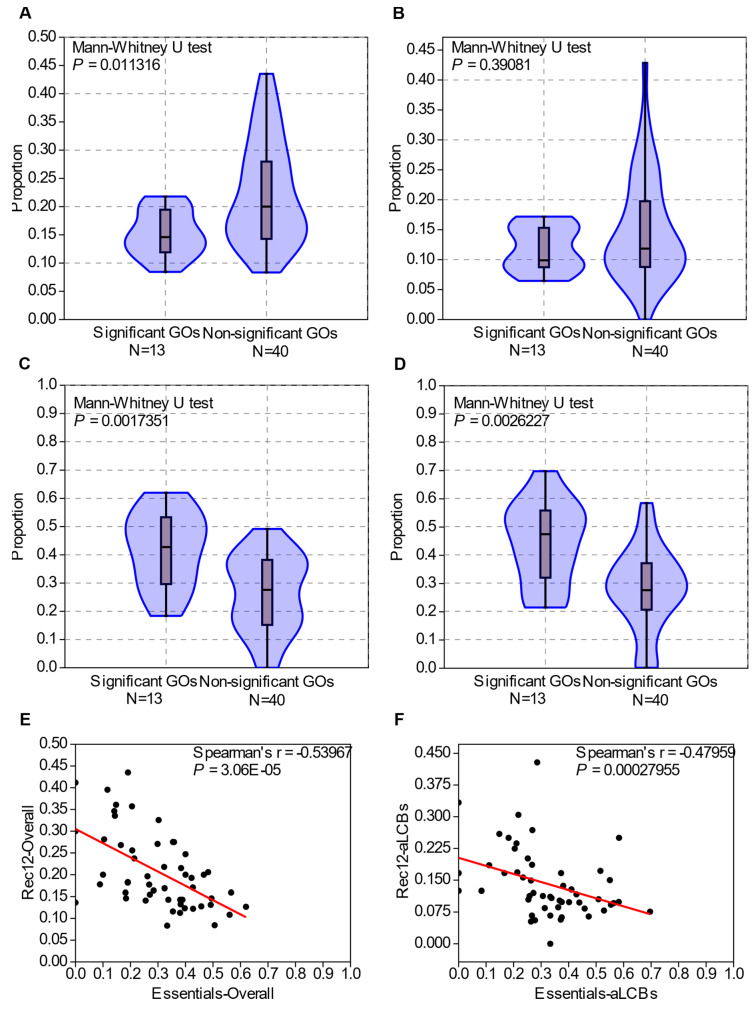
Density of Rec12 cleavage sites and of essential genes in the significant and non-significant GO categories. (**A**) Overall density of Rec12 sites in the 13 significant and 40 non-significant GO categories are significantly different. (**B**) Rec12 density only in the aLCBs proved to be not significantly different. (**C**,**D**) Density of essential genes in overall and in the aLCBs among the GO categories. There were significant discrepancies among the concerning values. (**E**,**F**) Correlation of the Rec12 site densities and essential gene densities. Black dots represent GO categories, red lines are regression lines. The data show moderate, but significant correlations.

First, we counted the number of genes which were located next to Rec12 cleavage sites in each GO slim terms and compared the data to the previous results (Supplementary Table S16). The percentages of the genes which had adjacent Rec12 sites were between 8% and 44% in the different GO categories. The 13 significant GO categories had an overall mean value (15%) lower than the others (23%) (Mann-Whitney U test, *p* = 0.011316) (Figure 7A). However, when we compared the Rec12 site abundance of the significant categories to the non-significant ones only in the aLCBs, then the difference was not significant (Mann-Whitney U test, *p* = 0.39081) (Figure 7B).

Then, we examined the density of essential genes in the GO terms and their localizations (Supplementary Table S17). The GO categories localised significantly to the aLCBs exhibited higher density of essential genes in overall (40% compared to 26%) and in the aLCBs (53% compared to 42%), too (Mann-Whitney U test, *p* = 0.0017351 and *p* = 0.0026227, respectively) (Figure 7C,D).

In the examination of the relationship between the proportion of Rec12 cleavage sites and the proportion of essential genes in the GO terms, we found a moderate, but significant relation (Spearman’s *rs* = −0.53957, *p* = 3.06 × 10^−5^) (Figure 7E). Surprisingly, the degree of correlation decreased when we considered only the genes located at the aLCBs (Spearman’s *rs* = −0.48959, *p* = 0.00027955) (Figure 7F).

## 4. Discussion

Our present study provided further evidence for the highly conserved gene content and order of the *Schizosaccharomyces* species. Orthology-based inference of collinear genome segments revealed the most conserved regions (aLCBs) of the fission yeast genomes and these segments are probably not just remnants of ancestral gene order but are also maintained by natural selection. These aLCBs have dispersed localisation on the chromosomes and might have been inherited from their last common ancestor, which was probably a filamentous fungus. The relevance of these findings and more are discussed below.

From a phylogenetic perspective, we showed that the fission yeasts exhibit an unequivocal correlation in the structural- and sequence evolution similarly to *Verticillium*, *Lachancea* and certain metazoan species [76,77,78,79,80]. However, the degree of correlation was unexpectedly high in spite of the great evolutionary divergence of the fission yeasts [2,5]. It is important to note that a former study with a different approach came to the same conclusion for the whole Taphrinomycotina subphylum [81]. Since most of the Taphrinomycotina species only inhabit or colonise a narrow range of niches, that highly specialised lifestyle of the concerned species might be the reason for the correlated evolution of their sequence and structure [2,39,82].

Another striking phenomenon was that extent of the conserved genomic regions of the four species was almost the same from the *S. japonicus* perspective. We found that 40–42% of the whole gene content located at aLCBs consisted of almost eight collinear genes on average. That was also remarkable considering that the human and fugu fish genomes share a lower sequence divergence overall than the fission yeasts do [2,5], but in the former pair only small groups of between two and three genes remained adjacent [83]. Besides, based on thorough examination of the dataset of [81], there is almost no genus in the Ascomycota phylum with the same sequence divergence who exhibit as highly conserved a gene order as the fission yeasts do.

The existence of syntenic gene pairs that are adjacent in many genomes could either be the consequence of selection or could have occurred by chance [66]. In the first scenario, selection may favour two genes being adjacent over large evolutionary distance to coordinate proper coregulation or co-expression, for instance. In the other case, gene pairs split, then become adjacent again due to the numerous rearrangements that possibly occur in a genome [66]. The analyses of our in silico models revealed that the existence of the aLCBs neither could be resulted by chance, nor were they just remnants of an ancestral gene order. These aLCBs were preserved even though a high number of chromosomal rearrangements occurred in the genomes. Our findings suggest that the inferred aLCBs might be under the control of maintaining selection.

Many hypothesized that ancestors of “modern” yeasts were filamentous fungi [5,84,85]. Further support for that view could be that the earliest diverging branch of the fission yeasts (*S. japonicus*) is a dimorphic species [18]. Here, we showed that the order of certain genes of the *Schizosaccharomyces* might also be reflected in the gene order of a filamentous ancestor. Although the gene order of the aLCBS seems to be under selection constraints, we have also demonstrated that sequences of the aLCBs are as mutable as any sequences in other parts of the genomes. These particular findings provide further evidence on the assumption that structural- and sequence evolution depend on different molecular clocks [73,86,87].

So, what could be the reason for the unusually stable genome structures of the fission yeasts? One possible reason could be the reproductive isolation caused by extensive rearrangements. Chromosomal rearrangements can lead to different chromosome sizes or structures, which have downside effects in the sexual cycles (e.g., improper pairing of chromatids in meiosis) [10,12,13,15]. Although new structural variants (SVs) could be advantageous in certain environments, the inefficient capability of producing viable offspring might be disadvantageous in the ever changing environment, especially in haplontic species [10,12,13,15]. Furthermore, several reports have provided evidence that sexual cycle fuels adaptation in different species better than spontaneous mutations or rearrangements do [17,88,89,90].

About 90% of the genome of *S. pombe* may contain functional elements [91], thus, the disruption of those might be disadvantageous in long evolutionary terms. Moreover, certain rearrangements can be deleterious and could lead to cell death [10,12,92]. Consequently, the occurrence of rearrangement events should be limited; thus, the gene order can be maintained. Our previous results also support this idea, as we showed that the *S. cerevisiae*-*S. uvarum* and the *S. cerevisiae*-*N. castelli* species pairs bore more chromosomal rearrangement events than the *S. octosporus*-*S. cryophilus* and the *S. pombe*-*S. cryophilus* pairs, which have almost the same divergence times [30].

Another reason might be that the adjacent genes constitute transcriptional and functional neighbourhoods which are common in higher eukaryotes [68,93,94,95], but interestingly, those segments are inclined to undergo rearrangements to create new functional units [96,97]. Thus, functional clustering does not necessarily explain long-term gene order stability. Besides, Tuller et al. failed to reveal such functional clusters in *S. pombe* [98]. Our results also support this view. Although we observed that groups of genes with certain biological functions (GO categories) tended to cluster to the aLCBs, it soon turned out that it was not the biological functions that were responsible for that. Analyses of the co-expression rates within and outside the aLCBs also support this, as we have not observed a significant difference between the co-expression values. The 3D conformation of the chromosomes might reveal functional clustering, but that topic is outside the scope of this article [99,100]. Instead, our data suggest that GO categories with a higher density of essential genes and with a lower abundance of Rec12 cleavage sites nearby are inclined to cluster to the aLCBs. These factors might also contribute to the conservation of the gene order, and, thereby, the maintenance of the genome structure.

Taken together, we suppose that several extrinsic (e.g., reproductive isolation, specialised lifestyle) and intrinsic (e.g., local density of essential genes) factors contribute to the maintenance of the genome structure and these factors may have stronger effects on the fission yeasts due to their haplontic state and lower number of chromosomes. However, the phenomena underlying the almost uniquely stable genome structures of the fission yeasts are still far from understood.

Nevertheless, revealing ancient collinear loci inherited from the last common ancestor provided us with a framework in which the acts of natural selection can be investigated. As future prospects, we would like to find answers to the following question: What could be the other determinants of gene order conservation in the genome of the fission yeasts? We also wish to know whether the aLCBs are “safe harbours” of the genomes for transgene integration or are under the constraints of purifying selection.

## Figures and Tables

**Table 1:** Comparison of the real and simulated values of the aLCB parameters. (**A**) The table show the differences between the real and the two distinct simulated datasets. (**B**) Statistical evaluations of the simulated datasets compared to the real values.

**(A)**	**Number of aLCBs**			**Number of Genes in aLCBs**			**Mean Number of Genes in aLCBs**		
**Real Data**	**266**			**2055**			**7.73**		
	**Min.**	**Max.**	**Mean**	**Min.**	**Max.**	**Mean**	**Min.**	**Max.**	**Mean**
Random evolution (*n* = 100)	5	23	13.12	33	140	77.75	5.09	7.4	5.93
ALF evolution (*n* = 100)	214	289	254.54	1387	1930	1656.44	6.15	6.9	6.5
**(B)**	**Number of aLCBs**			**Number of Genes in aLCBs**			**Mean Number of Genes in aLCBs**		
	**Real vs. Random**	**Real vs. ALF**		**Real vs. Random**	**Real vs. ALF**		**Real vs. Random**	**Real vs. ALF**	
Randomisation *p*	0.00990099	0.188118812		0.00990099	0.00990099		0.00990099	0.00990099	
Single-case *t*-probe *p*	1.53 × 10^−83^	0.44274		7.36 × 10^−95^	0.00016125		1.24 × 10^−5^	3.33 × 10^−14^	

**Table 2:** List of the 14 significant GO categories that are overrepresented in the aLCBs. The GO term meiotic nuclear division are not significant according to the single-case *t*-probe.

GO Slim Terms	Genes Found Overall	Genes in aLCBs	Genes in NCBs	Randomization *p*	Single-Case *t*-Probe *p*
Chromatin organization	275	128	147	0.019608	0.043888
Meiotic nuclear division	142	67	75	0.039216	0.072860
Mitochondrial gene expression	150	79	71	0.019608	0.010351
Mitotic sister chromatid segregation	182	91	91	0.019608	0.005432
mRNA metabolic process	281	140	141	0.019608	0.001176
Nucleocytoplasmic transport	109	64	45	0.019608	0.000021
Protein catabolic process	233	114	119	0.019608	0.009971
Protein modification by small protein conjugation or removal	185	89	96	0.019608	0.033979
Protein-containing complex assembly	262	122	140	0.019608	0.030717
Regulation of mitotic cell cycle phase transition	170	80	90	0.019608	0.040627
Ribosome biogenesis	323	156	167	0.019608	0.004532
Transcription, DNA-templated	459	205	254	0.039216	0.044262
tRNA metabolic process	166	93	73	0.019608	0.000877
Vesicle-mediated transport	319	152	167	0.019608	0.000801

**Table 3:** Comparison of the co-expression rates of genes from different regions of the genome. NCBs are for regions that are situated outside the aLCBs. * We handled the whole genome as one large block of genes.

	Number of Blocks	Number of Genes	Number of Co-Expression Cases	Mean Value of Co-Expression
In aLCBs	266	2055	7895	0.0408
In NCBs	232	1922	8175	0.0394
Whole genome	1 *	5063	12,814,452	0.0287

## Data Availability

All data generated or analysed during this study are included in this published article (and its Supplementary Information files). The custom Python scripts developed for this study are available at Github: https://github.com/Laci01/Laci01/tree/Schizosaccharomyces_synthetic, accessed on 13 October 2021.

## Supplementary Materials

The following are available online at https://www.mdpi.com/article/10.3390/jof7100864/s1, Supplementary File S1: Detailed description of the modelling processes. Figure S1: Pairwise whole genome alignments of the fission yeast species with the Mauve aligner; Figure S2: Statistical evaluation of the normalised data of amino acid differences, pairwise LCBs, pairwise MCDs and multiple MCDs; Figure S3: Pairwise whole genome dot plots of the fission yeast species created with YASS; Figure S4: Statistical evaluation of the pairwise whole genome dot plots; Figure S5: Histograms depicting the size distributions and frequencies of the found aLCBs; Figure S6: Pairwise Mauve alignments of the distantly related Taphrinomycotina species using *S. japonicus* as reference genome; Figure S7: Pairwise Mauve alignments of the distantly related Taphrinomycotina species using *S. pombe* as reference genome; Figure S8: Evolutionary rates of proteins whose genes are located at the aLCBS and the NCBs. Table S1: List of species used in this study; Table S2: Raw data of pairwise YASS alignment of *S. japonicus* and *S. pombe*, Table S3: Raw data of pairwise YASS alignment of *S. japonicus* and *S. cryophilus*; Table S4: Raw data of pairwise YASS alignment of *S. japonicus* and *S. octosporus*; Table S5: Filtered data of pairwise YASS alignment of *S. japonicus* and *S. pombe*; Table S6: Filtered data of pairwise YASS alignment of *S. japonicus* and *S. cryophilus*; Table S7: Filtered data of pairwise YASS alignment of *S. japonicus* and *S. octosporus*; Table S8: Descriptive statistics of the pairwise YASS alignments; Table S9: Putative orthologues of the fission yeasts; Table S10: List of aLCBs relative to the gene order of *S. pombe*; Table S11: List of putative orthologous genes of the chosen species regarding to the 11 random aLCBs; Table S12: List of the 26 common orthologous genes from the 14 species; Table S13: Pairwise Spearman correlation of the different parameters of the species; Table S14: Distribution of the genes from the main GO categories between aLCBs and NCBs; Table S15: Randomisation and statistical evaluation of the gene enrichments from the 31 candidate GO categories; Table S16: Rec12 cleavage site abundance in the aLCBs and in the NCBs; Table S17: Density of essential genes in the aLCBs and in the NCBs.
