# Supplementary material for: Genome Comparisons of the Fission Yeasts Reveal Ancient Collinear Loci Maintained by Natural Selection
Source: J Fungi (Basel). 2021 Oct 14;7(10):864. doi: 10.3390/jof7100864 (PMC8537764; doi:10.3390/jof7100864)
Supplement: Supplementary file 1 [file jof-07-00864-s001.zip › Supplementary figures.pptx]

## Slide 1
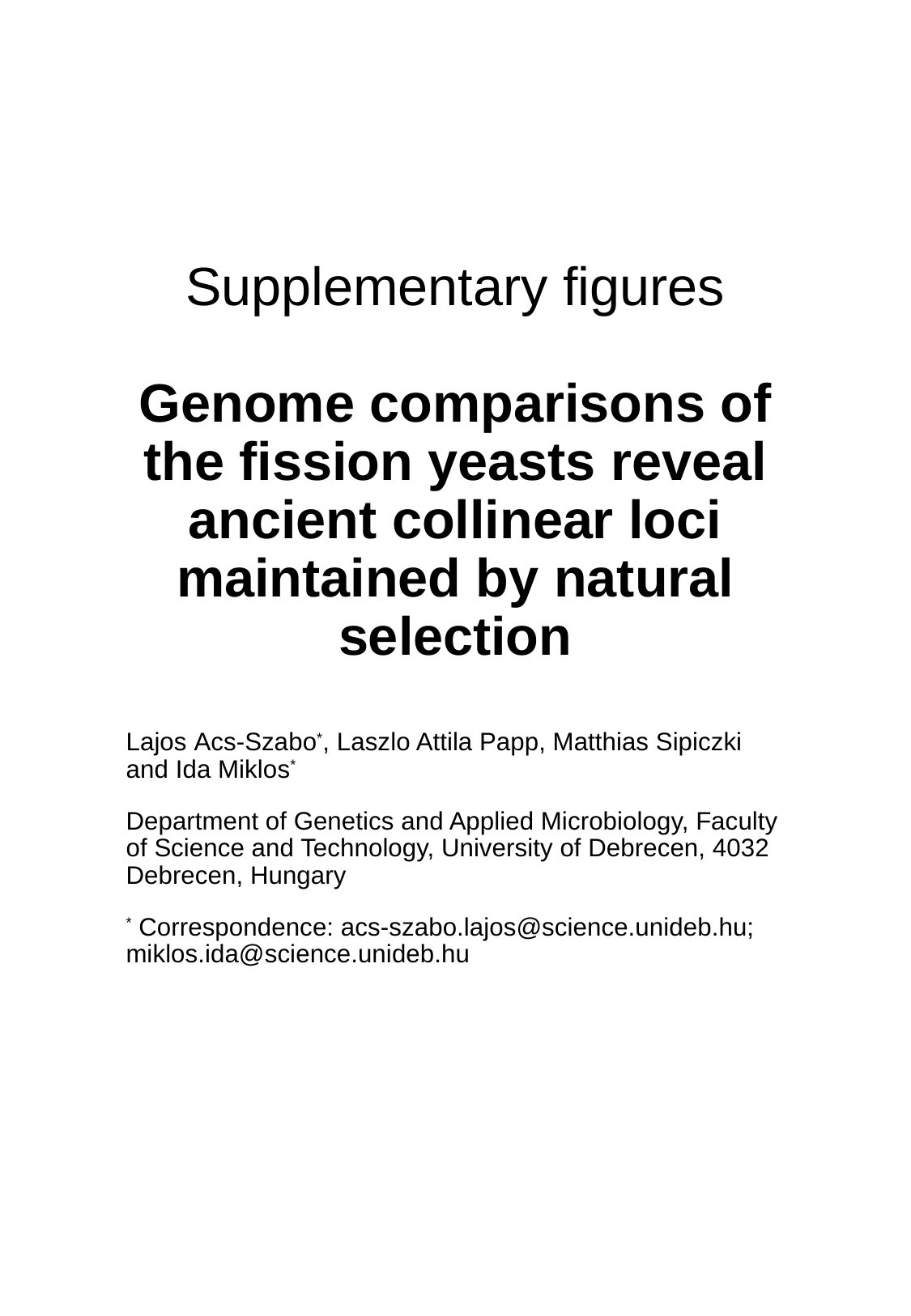

# Supplementary figures Genome comparisons of the fission yeasts reveal ancient collinear loci maintained by natural selection
Lajos Acs-Szabo*, Laszlo Attila Papp, Matthias Sipiczki and Ida Miklos*
Department of Genetics and Applied Microbiology, Faculty of Science and Technology, University of Debrecen, 4032 Debrecen, Hungary
* Correspondence: acs-szabo.lajos@science.unideb.hu; miklos.ida@science.unideb.hu

## Slide 2
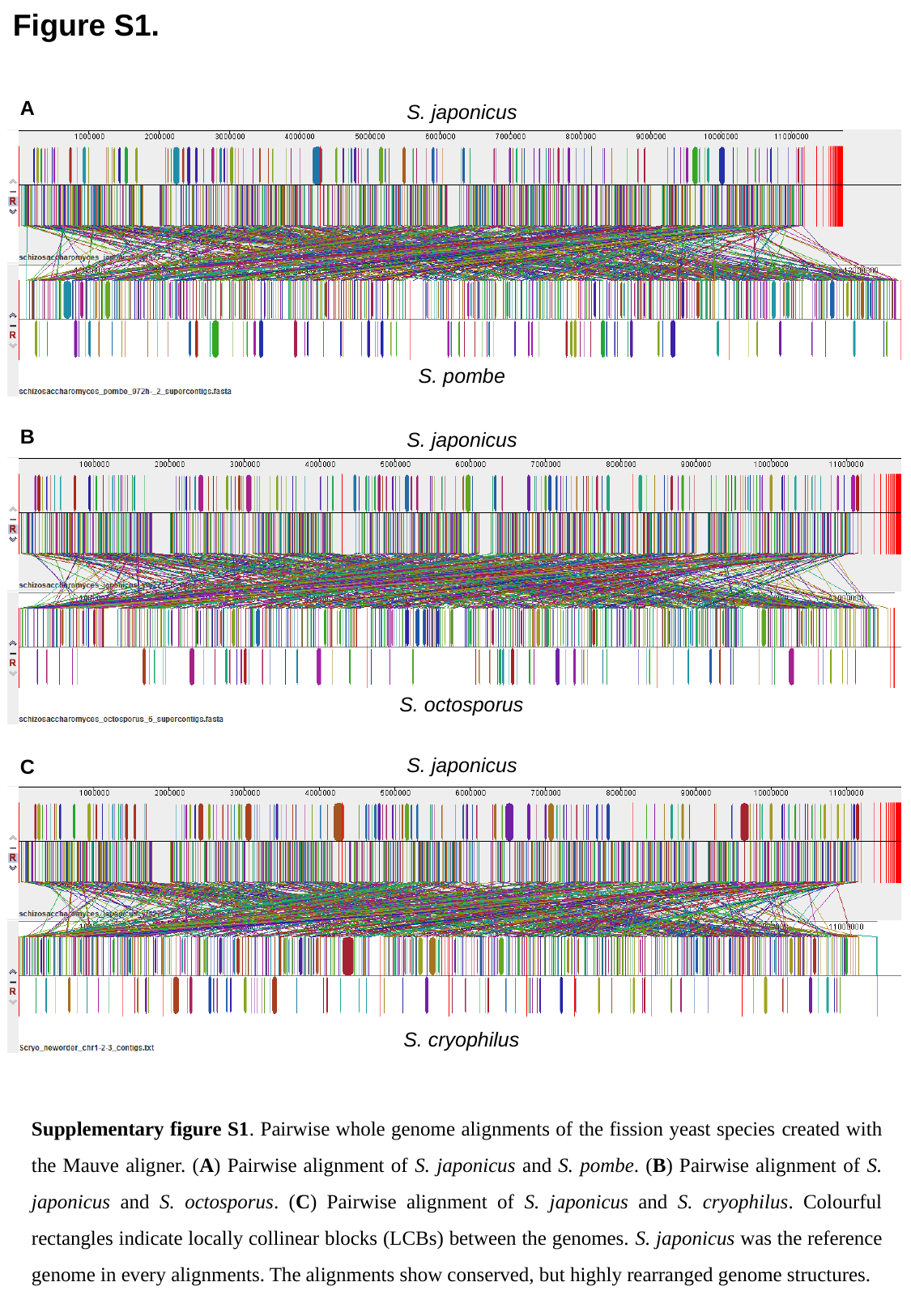

Figure S1.
A
S. japonicus
S. pombe
B
S. japonicus
S. octosporus
S. japonicus
C
S. cryophilus
Supplementary figure S1. Pairwise whole genome alignments of the fission yeast species created with the Mauve aligner. (A) Pairwise alignment of S. japonicus and S. pombe. (B) Pairwise alignment of S. japonicus and S. octosporus. (C) Pairwise alignment of S. japonicus and S. cryophilus. Colourful rectangles indicate locally collinear blocks (LCBs) between the genomes. S. japonicus was the reference genome in every alignments. The alignments show conserved, but highly rearranged genome structures.

## Slide 3
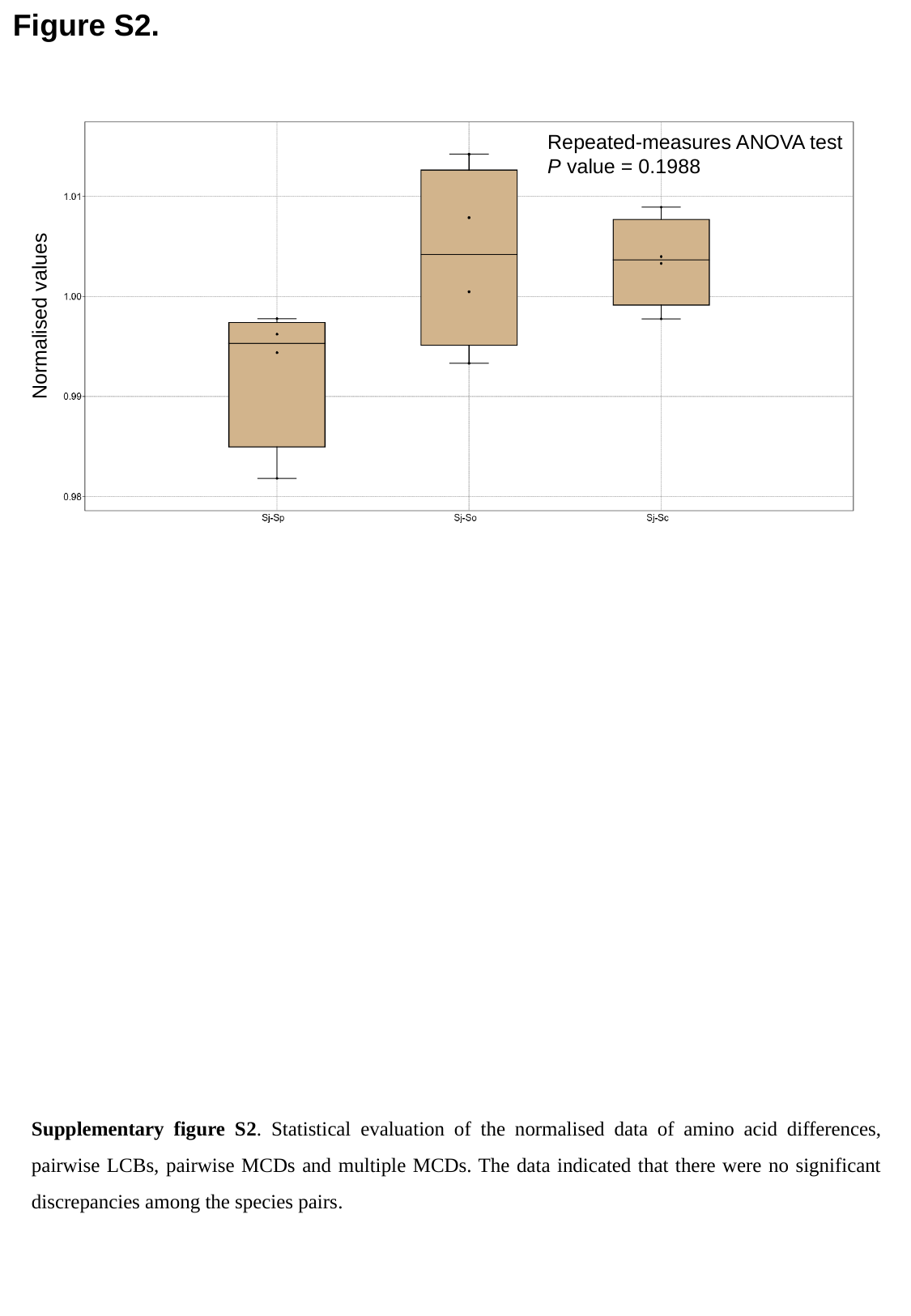

Figure S2.
Repeated-measures ANOVA test
P value = 0.1988
Normalised values
Supplementary figure S2. Statistical evaluation of the normalised data of amino acid differences, pairwise LCBs, pairwise MCDs and multiple MCDs. The data indicated that there were no significant discrepancies among the species pairs.

## Slide 4
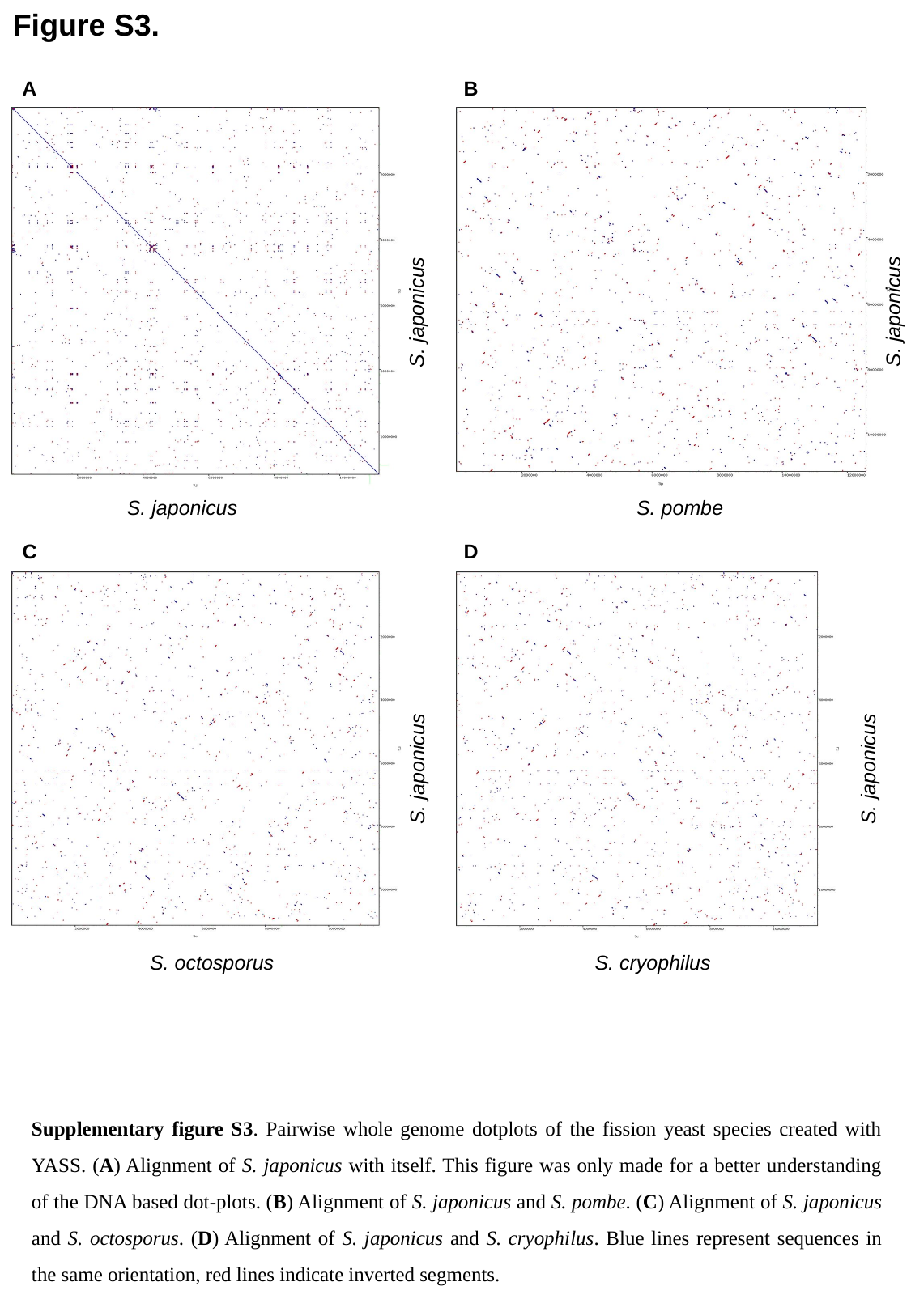

Figure S3.
A
B
S. japonicus
S. japonicus
S. japonicus
S. pombe
D
C
S. japonicus
S. japonicus
S. octosporus
S. cryophilus
Supplementary figure S3. Pairwise whole genome dotplots of the fission yeast species created with YASS. (A) Alignment of S. japonicus with itself. This figure was only made for a better understanding of the DNA based dot-plots. (B) Alignment of S. japonicus and S. pombe. (C) Alignment of S. japonicus and S. octosporus. (D) Alignment of S. japonicus and S. cryophilus. Blue lines represent sequences in the same orientation, red lines indicate inverted segments.

## Slide 5
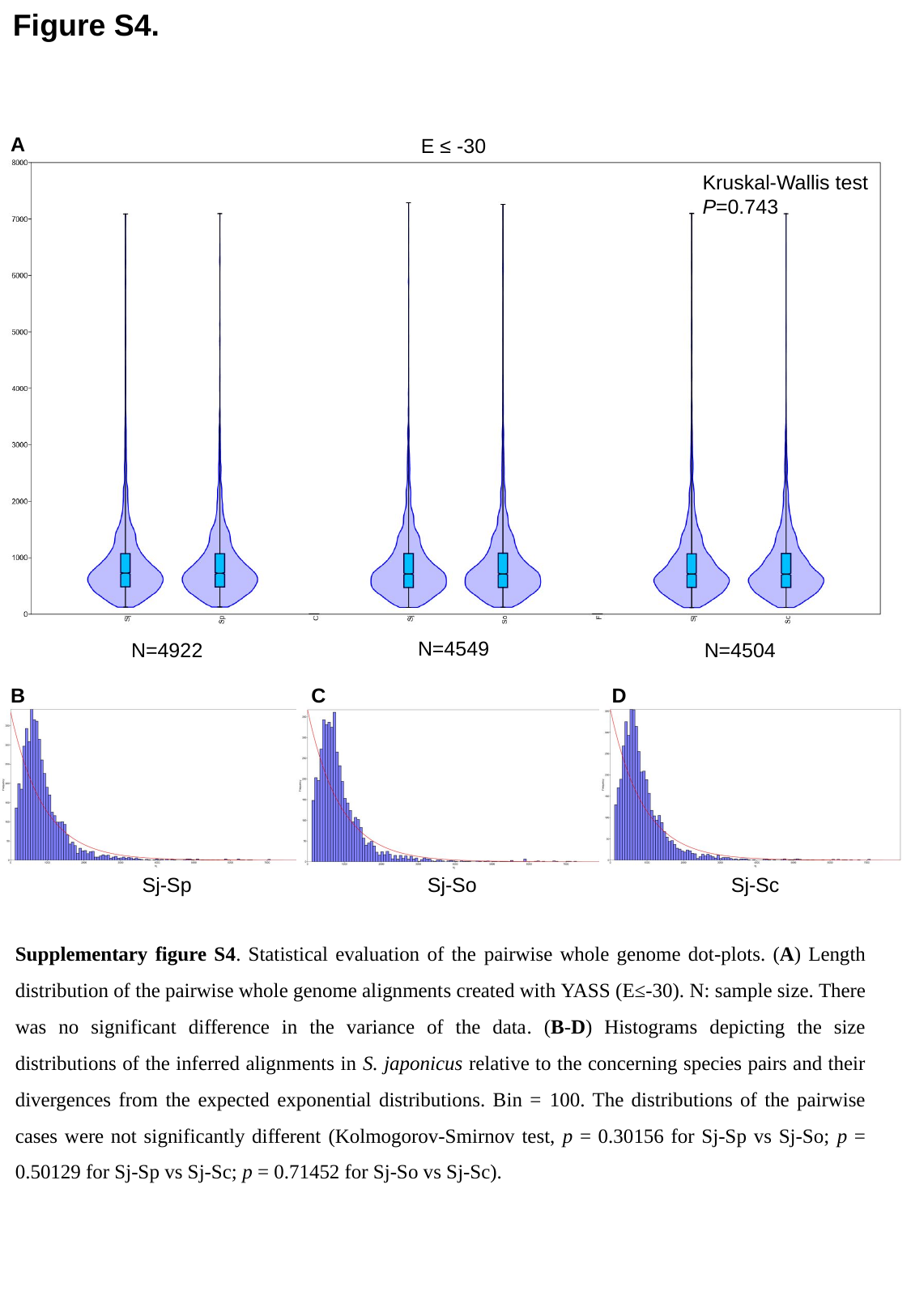

Figure S4.
A
E ≤ -30
Kruskal-Wallis test
P=0.743
N=4549
N=4922
N=4504
D
C
B
Sj-Sp
Sj-So
Sj-Sc
Supplementary figure S4. Statistical evaluation of the pairwise whole genome dot-plots. (A) Length distribution of the pairwise whole genome alignments created with YASS (E≤-30). N: sample size. There was no significant difference in the variance of the data. (B-D) Histograms depicting the size distributions of the inferred alignments in S. japonicus relative to the concerning species pairs and their divergences from the expected exponential distributions. Bin = 100. The distributions of the pairwise cases were not significantly different (Kolmogorov-Smirnov test, p = 0.30156 for Sj-Sp vs Sj-So; p = 0.50129 for Sj-Sp vs Sj-Sc; p = 0.71452 for Sj-So vs Sj-Sc).

## Slide 6
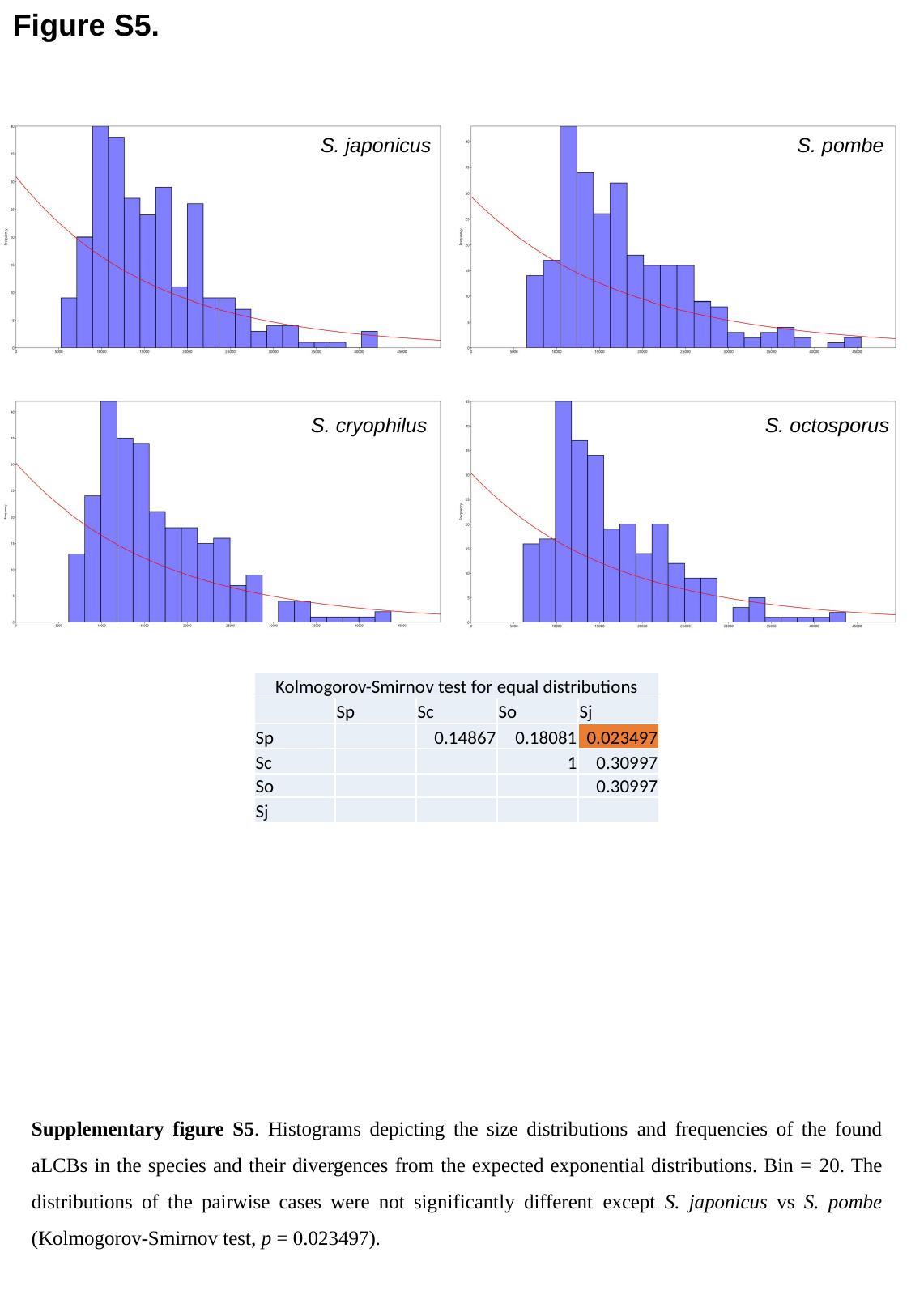

Figure S5.
S. japonicus
S. pombe
S. cryophilus
S. octosporus
| Kolmogorov-Smirnov test for equal distributions | | | | |
| --- | --- | --- | --- | --- |
| | Sp | Sc | So | Sj |
| Sp | | 0.14867 | 0.18081 | 0.023497 |
| Sc | | | 1 | 0.30997 |
| So | | | | 0.30997 |
| Sj | | | | |
Supplementary figure S5. Histograms depicting the size distributions and frequencies of the found aLCBs in the species and their divergences from the expected exponential distributions. Bin = 20. The distributions of the pairwise cases were not significantly different except S. japonicus vs S. pombe (Kolmogorov-Smirnov test, p = 0.023497).

## Slide 7
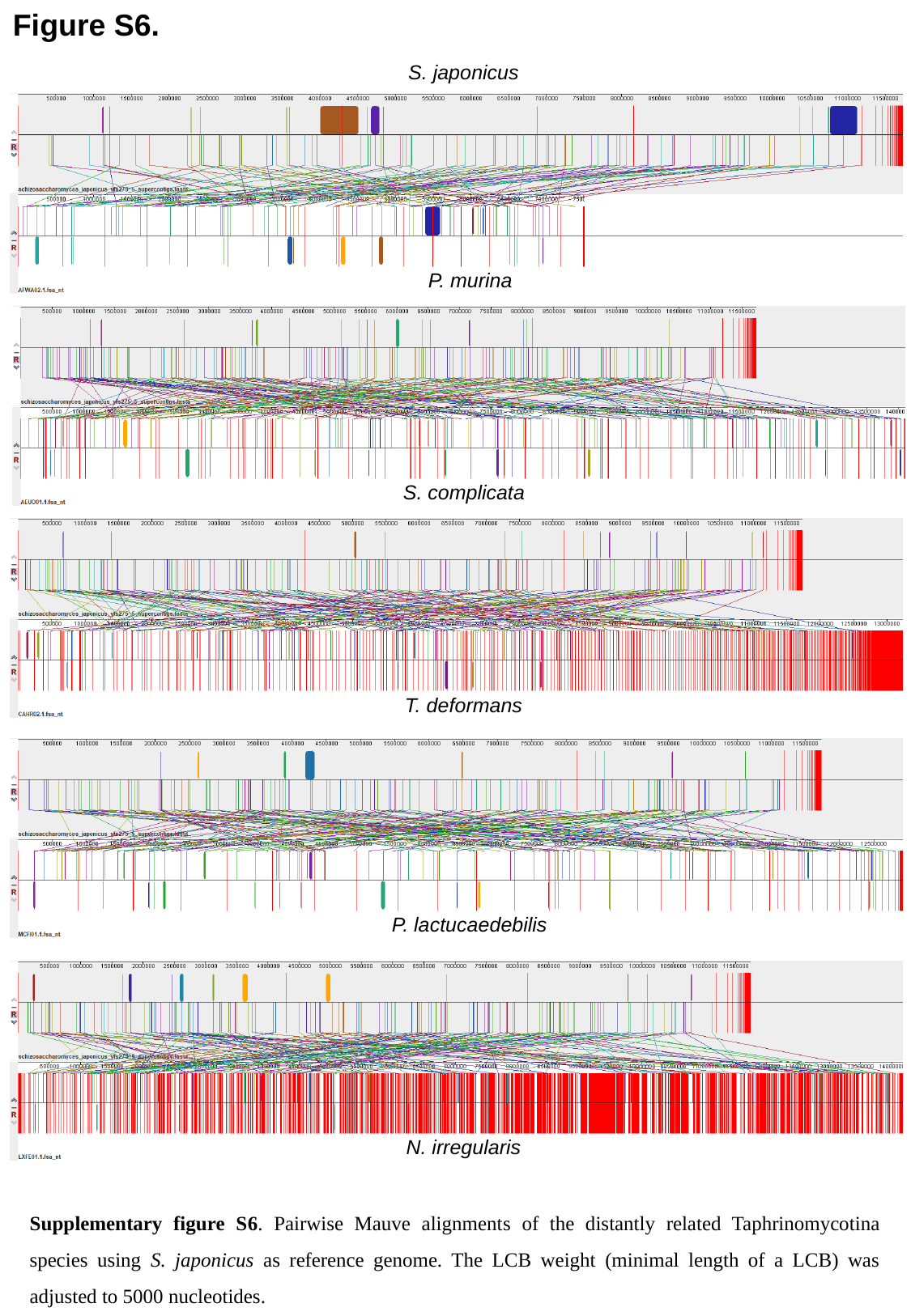

Figure S6.
S. japonicus
P. murina
S. complicata
T. deformans
P. lactucaedebilis
N. irregularis
Supplementary figure S6. Pairwise Mauve alignments of the distantly related Taphrinomycotina species using S. japonicus as reference genome. The LCB weight (minimal length of a LCB) was adjusted to 5000 nucleotides.

## Slide 8
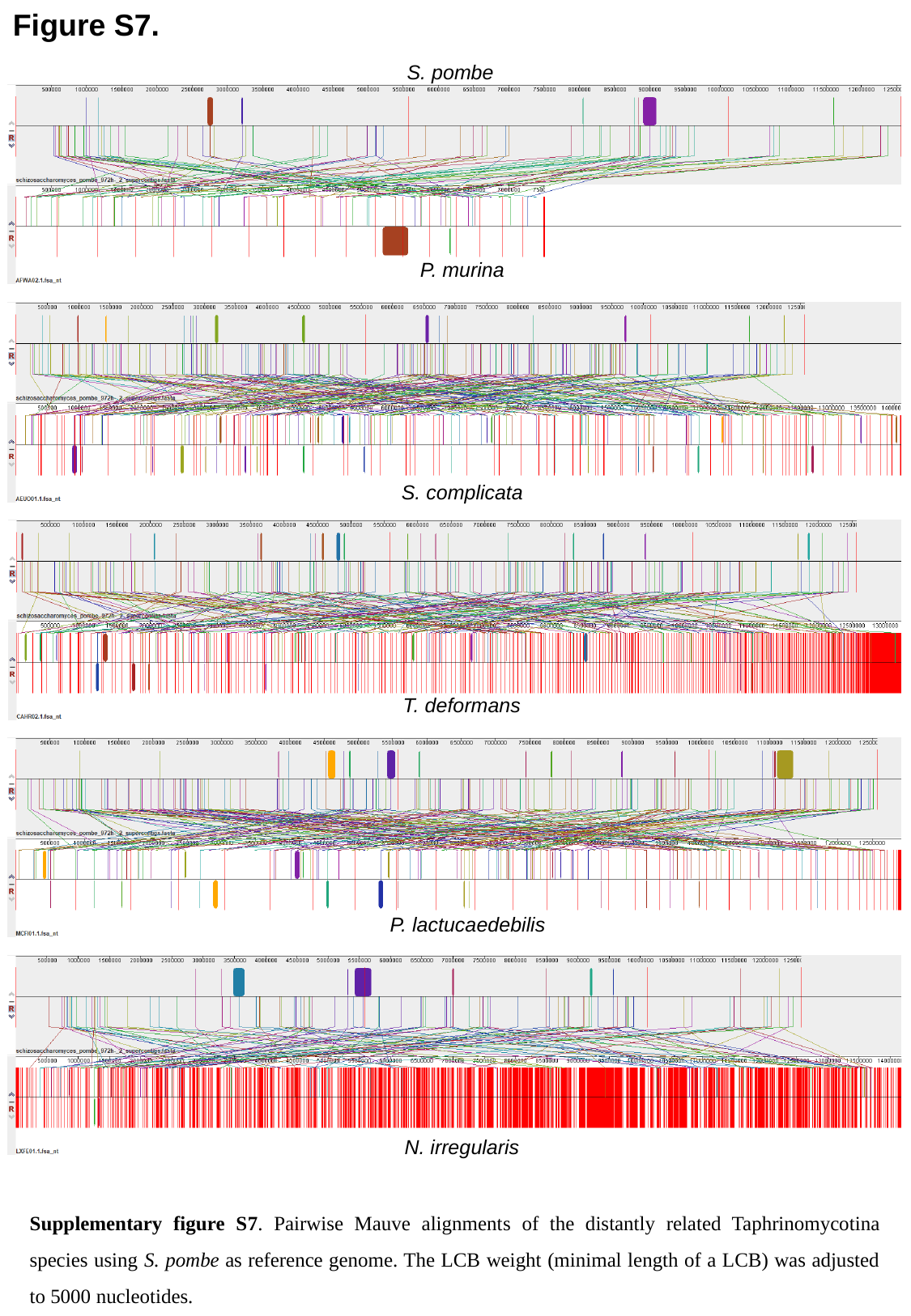

Figure S7.
S. pombe
P. murina
S. complicata
T. deformans
P. lactucaedebilis
N. irregularis
Supplementary figure S7. Pairwise Mauve alignments of the distantly related Taphrinomycotina species using S. pombe as reference genome. The LCB weight (minimal length of a LCB) was adjusted to 5000 nucleotides.

## Slide 9
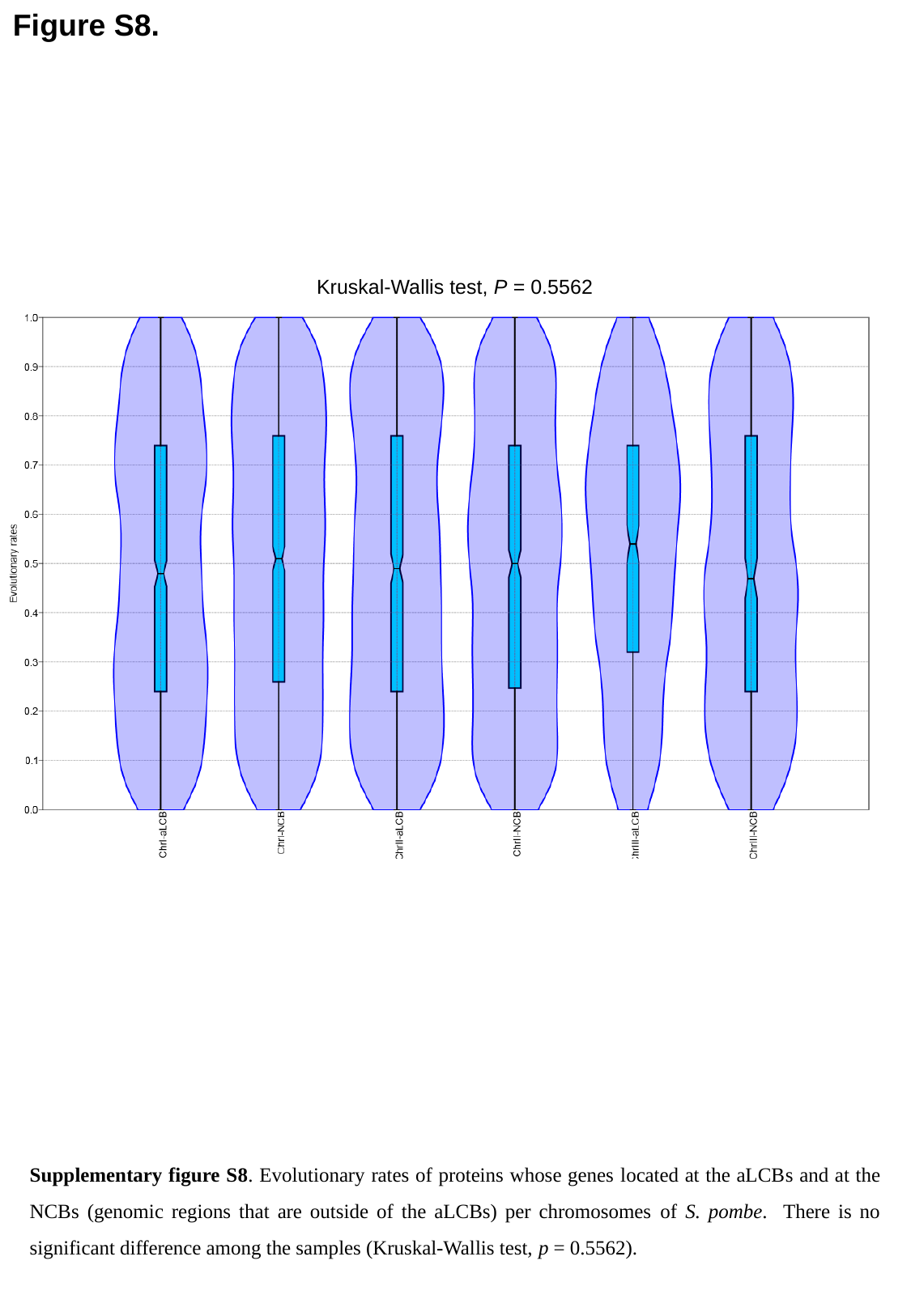

Figure S8.
Kruskal-Wallis test, P = 0.5562
Supplementary figure S8. Evolutionary rates of proteins whose genes located at the aLCBs and at the NCBs (genomic regions that are outside of the aLCBs) per chromosomes of S. pombe. There is no significant difference among the samples (Kruskal-Wallis test, p = 0.5562).
