# Supplementary material for: Genome Comparisons of the Fission Yeasts Reveal Ancient Collinear Loci Maintained by Natural Selection
Source: J Fungi (Basel). 2021 Oct 14;7(10):864. doi: 10.3390/jof7100864 (PMC8537764; doi:10.3390/jof7100864)
Supplement: Supplementary file 1 [file jof-07-00864-s001.zip › Supplementary File S1.pdf]

Supplementary information

Genome comparisons of the fission yeasts reveal ancient collinear loci maintained by natural selection

Lajos Acs-Szabo, Laszlo Attila Papp, Matthias Sipiczki and Ida Miklos
Department of Genetics and Applied Microbiology, Faculty of Science and Technology, University of Debrecen, Hungary

Correspondence: Lajos Acs-Szabo and Ida Miklos, Department of Genetics and Applied Microbiology, Faculty of Science and Technology, University of Debrecen, 4032 Debrecen, Egyetem tér 1., Hungary
E-mail: miklos.ida@science.unideb.hu
acs-szabo.lajos@science.unideb.hu

Foreword. The initial assumption of the simulations was that: if the genomes are evolving in a neutral way and are not under the control of some kind of selection, then the simulated data should be quite similar to that of observed in the real genomes. In order to ascertain that selection may have a role in the maintenance of the observed aLCBs, we performed a series of simulated genome evolutions with in-the-house built Python scripts and with the standalone version of the Artificial Life Framework (ALF) pipeline [65].

Detailed description of the synthetic genome evolution performed with the custom Python script. The script generates - simulated - random inversions without any selection pressure applied or considered. According to our recent knowledge, fission yeasts have 4878-5155 genes (it depends on the concerning species), so we use a root genome containing 5000 genes for the simulation. The script generates arrays from 1 to 5000 for *S. japonicus* as reference genome and for *S. pombe/S. octosporus/S. cryophilus* genomes as well. These numbers represent fictive genes on a theoretical chromosome.

This script uses numpy__version__ == 1.19.5

The randomization is based on the numpy.random.choice function where this function randomly selects 1 number (gene) from the array. Calling the choice function two times results a slice. The order of this slice then will be reversed and will replace the original order in the array. This will result 1 inversion event.

Calling the RandomGenomeGen with pombe_cycle=1, cryo_cycle=10, octo_cycle=50 does the following: it will generate 1 inversion in the case of *S. pombe*, 10 random inversions in *S. cryophilus* and 50 random inversions in *S. octosporus* (See figure below). The *S. japonicus* genome here is a reference genome which is unchanged and every changes are compared to its genomic state (See figure below).

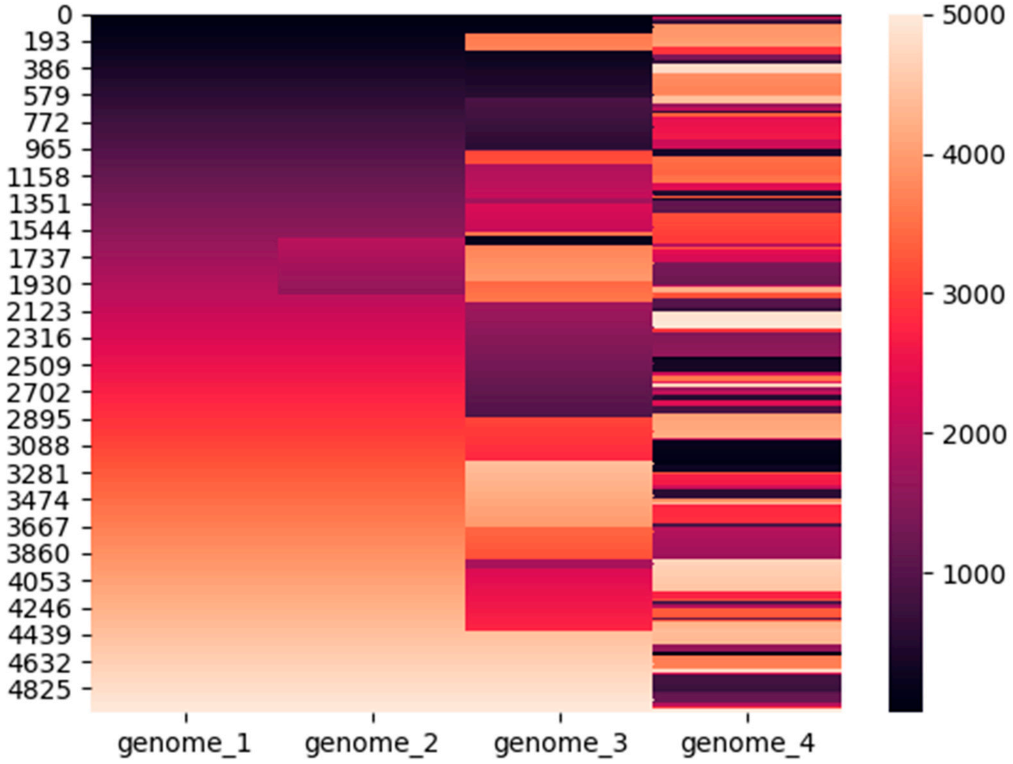

A heat map demonstrates the function of the custom Python script. Genome_1 represents the fictive *S. japonicus* genome, genome_2 represents the fictive *S. pombe* genome and so on. Numbers on the left side of the figure indicate the genes and their order in the reference genome. The script created certain number of random inversion at random sites in the fictive genomes: 1 inversion in genome_2, 10 inversions in genome_3 and 50 inversions in genome_4.

After the inversions in the genomes have been generated, the results will be printed out into .csv files where 5 columns can be found: an identifier column, the control (*S. japonicus*) column which remained unchanged and the transformed *S. pombe*, *S. cryophilus*, *S. octosporus* columns.

We use the previously estimated values of MCDs which came from the pairwise Mauve alignments followed by GRIMM rearrangement analyses. So, MCD values of *S. japonicus – S. pombe* = 590*; S. japonicus – S. cryophilus* = 592 and *S. japonicus – S. octosporus* = 598 were used for the 100 independent simulations.
For the analyses of the generated data, we use another custom script called Parser. This script searches for such aLCBs that we found in the real genomes. Namely, the script lists those cases where minimum 5 adjacent genes without any interruption can be found in the 4 synthetic genomes.

Detailed description of the synthetic genome evolution performed with the Artificial Life Framework (ALF). Although ALF designed to simulate the entire range of evolutionary forces that can act on a genome, we interested only in modelling molecular evolution in terms of genome rearrangements.

For the most realistic scenario, we had to specify several parameters for the ALF simulation. The exact parameters that were used for the simulation processes can be seen in the Materials and methods chapter in the main text, too. Here, we wanted to provide a detailed description on how the concerning parameters were defined exactly.

We used a root genome containing 5000 genes for the simulation as described in the previous chapter.

We also created a phylogenetic tree because ALF evolves a root genome along that given tree, where each node defines a speciation event. For the construction of the tree, we used the concatenated sequences of 18 specific proteins with evolutionary rates ranging from 0.02 to 0.52 [2]. The sequences were aligned with Muscle at the website https://www.ebi.ac.uk/Tools/msa/muscle/ [58,59] and filtered with Gblocks at http://molevol.cmima.csic.es/castresana/Gblocks_server.html [60]. For the phylogenetic tree construction we used the PhyML 3.0 algorithm available at http://www.atgc-montpellier.fr/phyml/ [61]. The phylogenetic inference based on 11159 well aligned sites. The number of substitution rate category was adjusted to 4, gamma distribution parameter was estimated and proportion of invariable sites was fixed to 0. Model selection for the analysis was done by SMS (http://www.atgc-montpellier.fr/phyml/) [62]. According to Akaike information criterion (AIC) and Bayesian information criterion (BIC) the LG model with +G +F decorations seemed to be the most suitable (AIC: 121072.63294; BIC: 121255.63298).

The topology of the constructed tree was concurred with the results of others [2,5,29]. For further conviction we compared the branch lengths of the tree to the amino acid

divergence of the fission yeasts established by [2] and the data were significantly correlated (Pearson's r = 0.95564, P = 0.002908). Hence, we supposed that the constructed phylogenetic tree will be a good starting point for the synthetic evolution.

Since we were concerned especially in genome rearrangements, the choice of sequence evolution models was minor. However, it was more important to establish a fission yeast specific rearrangement rate. In order to do that we took the number of rearrangements (inversion and translocations) into consideration which were estimated with GRIMM using the LCBs extracted from the Mauve alignments (see methods in the main text). Thus, we estimated the multi chromosomal distances (MCDs) in all 6 pairwise scenarios and divided the mean number of pairwise gene contents with them to create a pairwise-specific rearrangement rate. We used the mean value of the 6 rearrangement rates which was approximately 0.07 changes/genes.

It is also known that not only gross chromosomal changes like inversions and translocations but also gene duplications, gene losses and lateral gene transfers can significantly contribute to the loss of synteny in the genomes, thereby leaving these out from the analyses result underestimation of the changes possibly occurred. Therefore, we computed the mean values of gene gain, gene loss and gene duplication rates per genes using the concerning datasets of [2]. It is also important to note that these small scale changes were inferred only for the *S. pombe, S. cryophilus* and *S. octosporus* lineages relative to the *S. japonicus* lineage since we did not want to include the ancestor of *S. japonicus* because it would lead to an overestimation of rearrangement rates. Accordingly, the rates of gene gain, gene loss and gene duplication turned out to be 0.13, 0.05 and 0.01, respectively.

To simplify the modelling process, we added up the above values (0.07+0.13+0.05+0.01) and obtained an overall estimate for 0.26 changes/genes. This way, every type of changes are represented by inversions and translocations, so later we do not have to deal with new gene acquisitions and asymmetric gene losses in the inference of aLCBs after the simulations. Consequently, we applied a 0.13 rate for both inversions and translocations and we adjusted the rate of an inverted translocation to 0.5 as there is a 50% chance that a segment being translocated undergoes an inversion simultaneously. Although, the 0.5 rate for an inverted translocation is not seem to be realistic necessarily, we should bear in mind that ALF handle an individual genome as one large chromosome.

Finally, we adjusted the maximum length of rearrangements to 300 genes as this extent was the largest observable syntenic block between *S. pombe* and *S. octosporus*.

After the simulation processes were finished, we extracted the concerning information about the positions of the genes and analysed the results with the Parser script.
